# Estonian National Mental Health Study: Design and methods for a registry‐linked longitudinal survey

**DOI:** 10.1002/brb3.3106

**Published:** 2023-06-05

**Authors:** Kaia Laidra, Rainer Reile, Merle Havik, Mall Leinsalu, Carolina Murd, Jaan Tulviste, Merili Tamson, Kirsti Akkermann, Kairi Kreegipuu, Hedvig Sultson, Mare Ainsaar, Andero Uusberg, Jaana Rahno, Liisi Panov, Kadri Leetmaa, Anto Aasa, Toomas Veidebaum, Kelli Lehto, Kenn Konstabel

**Affiliations:** ^1^ Department of Epidemiology and Biostatistics National Institute for Health Development Tallinn Estonia; ^2^ Department of Chronic Diseases National Institute for Health Development Tallinn Estonia; ^3^ Stockholm Centre for Health and Social Change Södertörn University Huddinge Sweden; ^4^ Department of Drugs and Infectious Diseases Epidemiology National Institute for Health Development Tallinn Estonia; ^5^ Institute of Psychology University of Tartu Tartu Estonia; ^6^ Institute of Social Studies University of Tartu Tartu Estonia; ^7^ Department of Health Statistics National Institute for Health Development Tallinn Estonia; ^8^ Department of Geography University of Tartu Tartu Estonia; ^9^ Estonian Genome Centre, Institute of Genomics University of Tartu Tartu Estonia

**Keywords:** COVID‐19, mental health, population based, registry study, survey

## Abstract

**Objectives:**

The Estonian National Mental Health Study (EMHS) was conducted in 2021–2022 to provide population‐wide data on mental health in the context of COVID‐19 pandemic. The main objective of this paper is to describe the rationale, design, and methods of the EMHS and to evaluate the survey response.

**Methods:**

Regionally representative stratified random sample of 20,000 persons aged 15 years and older was drawn from the Estonian Population Register for the study. Persons aged 18 years and older at the time of the sampling were enrolled into three survey waves where they were invited to complete an online or postal questionnaire about mental well‐being and disorders, and behavioral, cognitive, and other risk factors. Persons younger than 18 years of age were invited to fill an anonymous online questionnaire starting from wave 2. To complement and validate survey data, data on socio‐demographic, health‐related, and environmental variables were collected from six national administrative databases and registries. Additionally, a subsample was enrolled into a validation study using ecological momentary assessment.

**Results:**

In total, 5636 adults participated in the survey wave 1, 3751 in wave 2, and 4744 in wave 3. Adjusted response rates were 30.6%, 21.1%, and 27.6%, respectively. Women and older age groups were more likely to respond. Throughout the three survey waves, a considerable share of adult respondents screened positive for depression (27.6%, 25.1%, and 25.6% in waves 1, 2, and 3, respectively). Women and young adults aged 18 to 29 years had the highest prevalence of depression symptoms.

**Conclusions:**

The registry‐linked longitudinal EMHS dataset comprises a rich and trustworthy data source to allow in‐depth analysis of mental health outcomes and their correlates among the Estonian population. The study serves as an evidence base for planning mental health policies and prevention measures for possible future crises.

## Graphical abstract

This paper describes the rationale, design, methods and survey response of the Estonian National Mental Health Study (EMHS), the first large‐scale population‐based mental health study in Estonia, conducted in 2021‐2022. The EMHS combined a three‐wave longitudinal survey with a registry‐based study and a validation study using ecological momentary assessments. The EMHS dataset comprises a rich and trustworthy data source to allow in‐depth analysis of mental health outcomes and their correlates among the Estonian population.

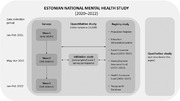

## 1 INTRODUCTION

According to the World Health Organization (2004), mental health is an integral part of health definition and can be understood as a state of well‐being that allows individuals to realize their abilities, cope with daily stressors and life events, work productively, and contribute to their community. The concept has further been clarified by Galderisi et al. (2015) as a dynamic state of internal equilibrium that rests on both cognitive and social skills and on the ability to recognize, express, and modulate one's emotions. Given its wide scope, there are a number of potential interlinked pathways how any imbalance in mental health could negatively affect health and well‐being. These may include (but are not limited to) the insecure home environment and early life experiences, problems with school completion, reduced career opportunities, financial stressors, and many other factors that can lead to unfavorable health outcomes during one's life course (Doran & Kinchin, 2019; Fekadu et al., 2019).

At the population level, mental health problems are associated with substantial disease burden, being among top 10 leading causes for loss of health due to either premature death or disease and disability (DALYs) (GBD 2019 Mental Disorders Collaborators, 2022). Moreover, the global number of DALYs due to 12 most frequent mental disorders has increased from 80.8 million in 1990 to 125.3 million in 2019, and its proportion from total disease burden increased from 3.1% to 4.9% (GBD 2019 Mental Disorders Collaborators, 2022). The public health impact of mental health problems can also be characterized by its considerable social and economic costs (Trautmann et al., 2016; Whiteford et al., 2013; Wittchen et al., 2011). Depending on the approach, the estimated global economic cost of mental health problems (based on data from 2010) varied between 2.5 and 8.5 trillion US dollars (Trautmann et al., 2016) and is expected to increase up to 16 trillion US dollars by 2030 (Bloom et al., 2011). This vast and growing societal impact emphasizes the urgency to tackle the mental health problems at the population level.

The role of mental health and well‐being in public health domain has become even more important during the ongoing COVID‐19 pandemic that in addition to its direct health impacts, has also created an environment where the existing risks for poor mental health are exacerbated. The pandemic has affected employment opportunities and educational engagement, financial security, access to health and social services in most societies, and thus re‐shaped our daily experiences. The mounting evidence on the mental health impact of COVID‐19 pandemic suggests increasing prevalence of stress, suicidal thoughts, anxiety, and depressive feelings (COVID‐19 Mental Disorders Collaborators, 2021; Fountoulakis et al., 2021; OECD], 2021; Reile et al., 2021; Wang et al., 2020). A recent meta‐analysis (COVID‐19 Mental Disorders Collaborators, 2021) estimated that COVID‐19 pandemic has resulted in additional 53.2 million cases of major depressive disorder and 76.2 million cases of anxiety disorders globally, increasing their prevalence by more than 25%. The increased prevalence of depression, post‐traumatic stress disorder (PTSD), anxiety symptoms, substance abuse, emotional disturbance, insomnia and avoidance behavior, and suicides associated with epidemic‐related anxiety and fear, has been reported also during earlier epidemics (Brooks et al., 2020; Cheung et al., 2008; Luo et al., 2020). Moreover, the duration of mental health impacts is not necessarily limited to the epidemic/pandemic period. Several studies have found long‐term increased risk of psychiatric disorders and suicides related to exposure to the SARS outbreak in 2003 (Liu et al., 2012; Mak et al., 2009; Tzeng et al., 2020; Wu et al., 2009, 2008). Given that similar long‐term consequences on mental health have also been found for economic crises (Frasquilho et al., 2016) and other natural or man‐made disasters (Norris et al., 2002), the detailed and timely data collection is paramount to adequately assess the public health impact of the pandemic.

Although in Estonia mental health became prominent in public discourse already before COVID‐19 pandemic, only a few policy documents have been published so far. The 2020 Green Paper on Mental Health (Sotsiaalministeerium, 2020) emphasized the need to enhance the prevention and early detection of mental health problems and the availability of high‐quality care. However, up to this point, there is no monitoring system to assess the prevalence of mental health problems and treatment needs within general population. Although mental health indicators have been included in several regular surveys conducted in Estonia (e.g., Health Behaviour in School‐Aged Children, Estonian Health Interview Survey, Survey of Health, Ageing and Retirement in Europe, and Health Behaviour Study Among Estonian Adult Population), neither of those allows a detailed assessment of population mental health. This is problematic as surveillance of population mental health and identification of vulnerable groups is essential for mental health policy planning and prevention. The COVID‐19 pandemic not only exacerbated the need for a thorough overview of the population mental health situation in current crisis but also highlighted the necessity to increase the preparedness for any future crises.

This paper presents an outline of the rationale, design, and methods of the Estonian National Mental Health Study (EMHS) and evaluates the survey response. The EMHS is the first large‐scale population‐based study focusing on mental health in Estonia and it aimed to: (a) provide a comprehensive overview of the mental health and related lifestyle factors of the Estonian population during the ongoing COVID‐19 pandemic, (b) explore the mechanisms behind the development of mental health issues in stressful situations, (c) evaluate the need for national support services in a crisis, and (d) propose a set of survey and registry‐based indicators that could be used for nationwide mental health monitoring. The study was commissioned by the Estonian government to gain an overview of the population mental health and to prepare the ground for building up a mental health monitoring system for Estonia. The EMHS was conducted by researchers from the Estonian National Institute for Health Development (NIHD) and University of Tartu.

## 2 METHODS

### 2.1 Study design and timeline

**FIGURE 1:**
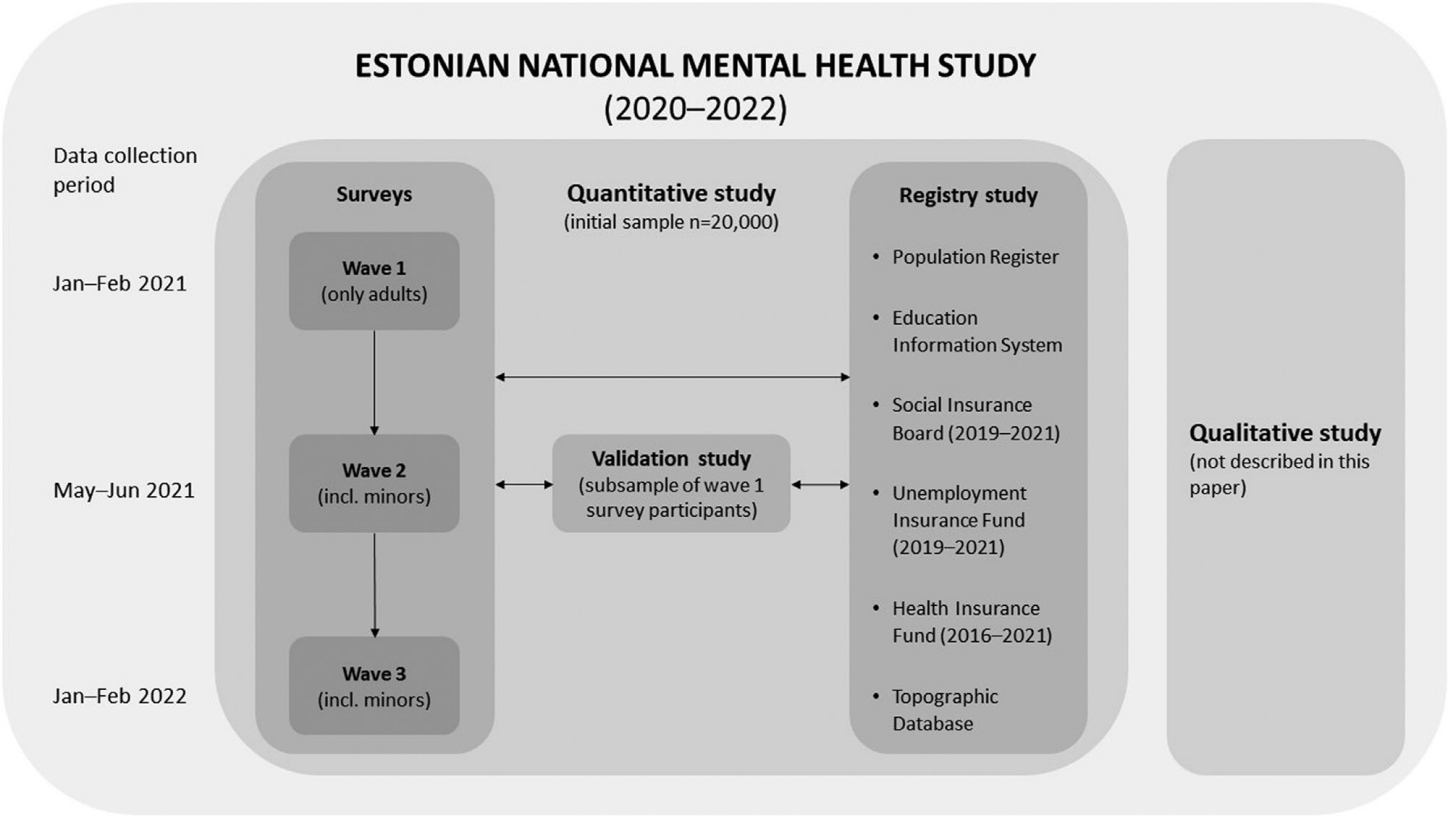
The structure and timeline of the EMHS.

The EMHS is a methodologically complex study that combined: (a) repeated cross‐sectional and longitudinal surveys, (b) registry‐linked study, (c) validation studies, and (d) qualitative studies. The core elements of the EMHS were population‐based repeated cross‐sectional surveys and registry study. The surveys were carried out in three waves to allow temporal comparisons from both cross‐sectional and longitudinal perspectives. Registry data, covering the period between 2016 and 2021, were gathered from six national databases and were individually linked to survey data. To validate the self‐assessed indicators, an ecological momentary assessment study with sleep and physical activity study components was conducted on a subsample of survey participants. Because qualitative studies were conducted independently and were based on a separate sample, they remain out of the scope of the present paper. The EMHS was conducted between January 2021 and February 2022. The structure and timeline of the EMHS are compactly shown in Figure 1.

### 2.2 Study sample

The sample frame for the EMHS consisted of 1,110,274 permanent residents of Estonia aged 15 years and older according to the Population Register data as of January 1, 2020 (Statistics Estonia). A stratified systematic random sampling was used to select 20,000 individuals from the sample frame. The sample size was predetermined to ensure enough respondents for (a) region‐specific comparisons, and (b) three‐wave longitudinal analyses at the population level. The sample frame was first divided into 17 non‐overlapping regional strata (15 counties and two largest cities—Tallinn and Tartu). Based on sample size calculations (alpha = 0.05, power = 80%) and modelling of different response rate scenarios (response rates per survey wave 25%−40%), the initial sample size for each regional stratum was defined as 1000 individuals (*n* = 17,000). Within each regional stratum, 30 strata were formed by sex and 5‐year age groups (15−19, …, 85+) proportional to the distribution of the target population in the region, resulting in 510 strata.

As previous population‐based health surveys in Estonia have demonstrated systematically lower participation rates for men and those in younger age groups (Reile & Veideman, 2021), different age‐ and sex‐specific inclusion probabilities were used to guarantee enough participants in each stratum. Applying oversampling coefficients (ranging from 1.1 to 1.5), additional 3000 cases were distributed to age‐ and sex‐specific strata resulting in the total sample size of 20,000 individuals with the mean size of a regional stratum being 1176.

To obtain the sample, a formal application (approved on November 24, 2020) was sent to the Estonian Ministry of the Interior that administers the Population Register database. Sampling was carried out at the Population Register according to the sampling procedures described above. Sample information for each individual included personal identification number, forename and surname, date of birth, sex, registered postal address, mobile phone number and email address(es), information about citizenship, native language, and ethnicity. These personal data were necessary for surveys (for sending personalized survey invitations) and the registry study (for making queries to other registries/administrative databases). Data on legal marital status and highest completed level of education were additionally requested for the registry study.

### 2.3 Surveys

#### 2.3.1 Procedures

##### Timeline

The surveys were carried out in mixed mode (web and postal survey) in three 2‐month data collection waves between January 2021 and February 2022. The first wave was started on January 4, 2021, the second on May 3, 2021, and the third wave on January 3, 2022.

##### Eligibility criteria

**FIGURE 2:**
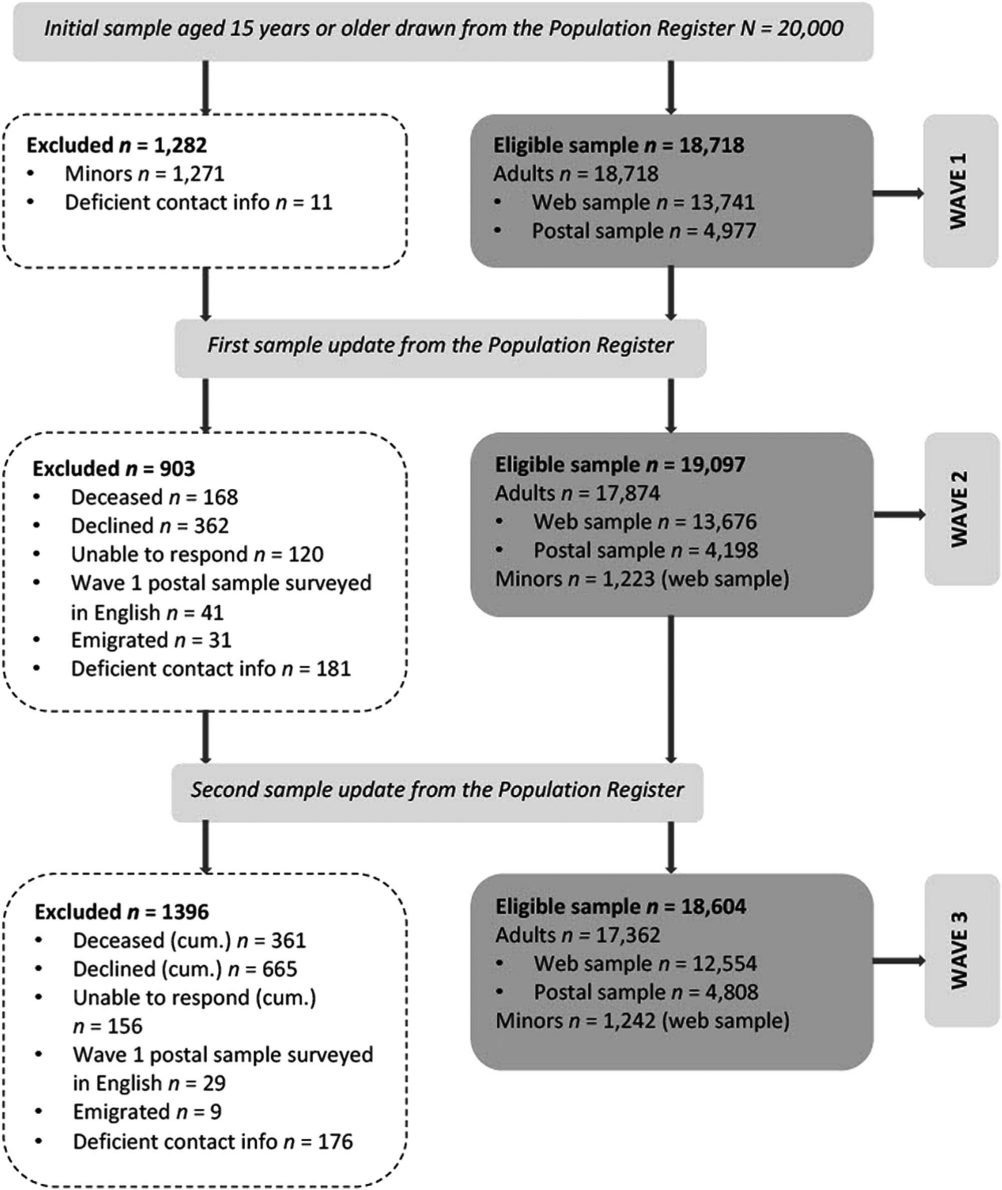
Formation of eligible survey sample from wave 1 to wave 3.

The eligibility of sampled individuals into each survey wave was based on: (a) age criteria, (b) status of residence, (c) availability of contact data, and (d) informed consent (see Figure 2). Persons aged 15−17 years at the time of sampling (*n* = 1271) were recruited anonymously (without possibility for individual record linkages) only to the second and third survey wave because of legal restrictions regarding surveying minors (*Isikuandmete kaitse seadus* [Personal Data Protection Act], 2019) and time pressure to start wave 1 data collection. Before the second and third survey wave, the status and contact information of the sample was updated after the query to Population Register and individuals who had deceased or emigrated from Estonia in between the waves were removed from the contact list. Participation in the study was voluntary and based on informed consent. Individuals who notified the organizers of their wish to not participate or withdraw their consent were not contacted in subsequent waves and their data were deleted from the database (except from the file with initial sample data). Individuals who were not able to respond due to a persistent medical or other impeding condition were removed from the contact list in the subsequent wave(s), but their data were kept in the database.

##### Recruitment

Based on the contact data of the sample, individuals were assigned to either web (at least one valid email address available) or postal (no email address given) subsamples and given personal study numbers. For the web subsample, a personalized email invitation containing the direct link to the survey in the LimeSurvey (LimeSurvey GmbH, Hamburg, Germany; http://www.limesurvey.org) environment was sent via Smaily email marketing and automation platform (Sendsmaily OÜ, Tallinn, Estonia; https://smaily.com). Information about the study was briefly presented in the invitation email and in more detail on the opening page of the web survey. At the end of the questionnaire, participants were provided a list of useful links and recommendations in case they wished to obtain further information about mental health or to consult someone about their mental health.

A random sample of participants (*n* = 1000) from the web subsample, who had not completed the survey in waves 1 and 2, were assigned to the postal subsample in wave 3. This will allow to assess the effect of recruitment mode on participation.

Persons in the web subsample who had neither completed the questionnaire during the designated period nor declined to participate, were sent up to three reminders by email and one reminder by short message service (SMS) to those with mobile phone number available. The SMS was sent via Direct Messenger SMS platform (Direct Messenger OÜ, Tallinn, Estonia; https://messenger.ee/) to the persons who had neither opened the emails nor followed the link to the LimeSurvey platform. The time between email reminders was approximately 7 days. For anonymous survey of minors aged 15−17 in waves 2 and 3, anonymous mode of the LimeSurvey was used, where information on whether a specific person has completed the questionnaire is available, but identification of individuals from the data is not possible.

For postal subsample, printed survey materials (a cover/informed consent letter with information about the study, a questionnaire, a pre‐paid envelope to return the questionnaire, and an information leaflet with mental health support contacts) were sent to person's home address. The postal subsample had also the possibility to complete the questionnaire online by following the link on the study website and entering the personal study number marked on the printed questionnaire. In case the completed questionnaire (either on paper or on web) was not returned within 2 weeks from the initial posting, a reminder was sent by post. Four weeks after the initial posting, another full set of survey materials was sent to non‐responders.

The survey was conducted in three languages (Estonian, Russian, and English) in the first wave and in two languages (Estonian and Russian) in the later waves to optimize the costs of the study. The survey language for each person was determined based on the reported ethnicity and/or native language and/or official citizenship in the Population Registry. In the online survey, participants had the opportunity to choose the language of their preference. In postal survey, participants could change the survey language by contacting the organizers. In waves 2 and 3, the web subsample initially assigned to the English version was contacted in Estonian (the official language of Estonia) and they could use automatic translation into English. The postal subsample assigned to the English version in wave 1 was not recontacted in subsequent waves, because the expected response rate for mailing the Estonian version was considered too low relative to the costs. However, if a valid email address became available via sample update (applicable also for the rest of the sample), these persons were included in the web subsample in subsequent waves.

##### Incentives to motivate participation

To motivate participation, 50 supermarket gift cards worth 30 Euros each were drawn between the respondents after every survey wave. After the third wave, additional 20 gift cards worth 100 Euros were drawn between the participants who responded in all three survey waves.

#### 2.3.2 Measures

Based on the study objectives, the following topics were covered in the survey: (a) socio‐demographic information, (b) indicators of mental well‐being and disorders, (c) risk and protective factors for mental health, (d) COVID‐19‐specific stressors, and (e) mental health support. For each topic, previously validated measures and items (if available) were considered first to enable comparisons with other studies. However, shorter instruments were often preferred or a few items from longer instruments were selected to limit the average completion time of the survey to less than 30 min. If needed, available measures were modified to better meet the needs of the present study (e.g., response scales and time frames of similar measures were harmonized). Also, several new items were developed specially for the EMHS.

The questionnaires for adults consisted of core measures/items that were repeated in all three waves to obtain a longitudinal perspective and other measures/items that varied by survey wave. The core items included the most important mental health indicators and their risk factors, as well as main socio‐demographics. Items that varied by wave measured more stable constructs that were less likely to change during the study period. The block of the COVID‐19‐related stressors was updated for each wave according to the current state of the epidemic. An overview of the study variables and measures is presented in Table 1. Complete questionnaires for adults can be found in Additional files 1−3.

**TABLE 1:** Overview of constructs and measures by subsample and survey wave

		Adults			Minors	
Variable or construct	Measure	W1	W2	W3	W2	W3
*Socio‐demographic background*						
Gender		x	x	x	x	x
Date of birth/age^c^		x	x	x	x	x
Ethnicity		x	–	–	x	x
Main language at home		x	–	–	x	x
Current marital status		x	x^a^	x	–	–
Highest level of education		x	x^a^ ^b^	x	–	–
Current employment status		x	x^a^	x	–	–
Study status^c^		x	x^a^	x	x	x
Occupation		x	x^a^	x	–	–
Monthly net income		x	x^a^	x	–	–
Current financial situation^c^		x	x^a^	x	x	x
Household composition^c^		x	x^a^	x	x	x
Need for constant care in the household		x	x^a^	x	–	–
Number of rooms used by the household		x	x^a^	–	–	–
Having a private room/space		–	–	–	x	x
Current place of residence		x	x^a^	x	x	x
Type of settlement		x	x^a^	x	x	x
Residing in officially registered address		–	x	–	–	–
Types of immediate neighborhood		–	x	–	–	–
Disturbing factors in daily environment		–	x	–	–	–
Living with pets		–	x	–	–	–
B. *Mental health*						
Satisfaction with aspects of life^c^		x	x	x	x	x
Diagnosed with a mental disorder^d^		x	x	x	–	–
Attention deficit/hyperactivity disorder	Modified Estonian version of the Adult ADHD Self‐Report Scale‐V1.1 Screener (Kessler et al., 2005)	x	–	–	x	x
Depression	Emotional State Questionnaire 2 (EST‐Q2; Aluoja, Shlik, Vasar, Luuk & Leinsalu, 1999)	x	x	x	x	x
Anxiety	EST‐Q2 (Aluoja et al., 1999)	x	x	x	x	x
Agoraphobia–panic	ESQ‐2 (Aluoja et al., 1999)	x	x	x	x	x
Fatigue	EST‐Q2 (Aluoja et al., 1999)	x	x	x	x	x
Insomnia	EST‐Q2 (Aluoja et al., 1999)	x	x	x	x	x
Intentional self‐harm		x	x	x	x	x
Mania	DSM‐5 Self‐Rated Level 1 Cross‐Cutting Symptom Measure—Adult (DSM‐5 Screener; American Psychiatric Association [APA], 2013)	x	x	x	x	x
Somatic symptoms	DSM‐5 Screener (APA, 2013)	x	x	x	x	x
Psychosis	DSM‐5 Screener (APA, 2013)	x	x	x	x	x
Memory	DSM‐5 Screener (APA, 2013)	x	x	x	x	x
Repetitive thoughts and behaviors	DSM‐5 Screener (APA, 2013)	x	x	x	x	x
Dissociation	DSM‐5 Screener (APA, 2013)	x	x	x	x	x
Mental well‐being		x	x	x	x	x
General trauma exposure^d^	Exposure item from the Primary Care PTSD Screen for DSM‐5 (Prins et al., 2016)	x	x	x	x	x
Specific childhood trauma exposure	Adapted from the Adverse Childhood Experience Questionnaire for Adults (Felitti et al., 1998)	–	–	x	–	–
Specific adulthood trauma exposure		–	–	x	–	–
Post‐traumatic stress disorder	Three items from the PTSD Checklist—Civilian Version (Weathers et al., 1993)	x	x	x	x	x
Eating disorders	Selected items from *Söömishäirete hindamise skaala* [Eating Disorders Assessment Scale] (Akkermann et al., 2010)	x	x	x	x	x
Gambling disorders	Brief Biosocial Gambling Screen (Gebauer et al., 2010)	x	–	–	–	–
Autistic traits	Ten items from the Autism‐Spectrum Quotient (Baron‐Cohen et al., 2001)	–	–	x	–	x
C. *Risk and protective factors*						
Height		x	–	x	x	x
Weight		x	–	x	x	x
Health insurance		x	–	–	–	–
Self‐rated health		x	x	x	x	x
Longstanding health problem		x	–	x	x	x
Concerned about health		x	x	x	x	x
Health‐related quality of life	EQ‐5D‐3L (EuroQol Group, 1990)	x	–	x	–	–
Physical activity^c^ ^d^		x	x	x	x	x
Current and past smoking		x	x	x	x	x
Hazardous alcohol use	Alcohol Use Disorders Identification Test (AUDIT; Babor et al., 2001)	x	x	x	x	x
Alcohol dependence	AUDIT (Babor et al., 2001)	–	x	–	–	–
Harmful alcohol use	AUDIT (Babor et al., 2001)	–	x	–	–	–
Use of narcotic substances	Adapted from the DSM‐5 Screener (APA, 2013)	x	x	x	x	x
Hours slept a day		x	x	x	x	x
Daily portions of fruits, vegetables, sweets, salty snacks, and sugary drinks		–	x	–	–	–
Coping with stress	One item from the of the Perceived Stress Scale (Cohen et al., 1983) Estonian version (Kallasmaa, 1997)	x	–	x	x	x
Emotionally demanding work	One item adapted from the Copenhagen Psychosocial Questionnaire III (Burr et al., 2019)	x	–	–	–	–
Emotion regulation	Three items from the Difficulties in Emotion Regulation Scale (Gratz & Roemer, 2004)	x	–	–	x	x
Cognitive appraisal		x	–	–	–	–
Perceived social support^c^, ^d^		x	–	–	x	x
Impact of pandemic on close relationships		–	x	–	–	–
Emotional abuse		x	x	x	x	x
Domestic abuse^d^		x	x	x	–	–
Sexual abuse		–	–	–	x	x
Physical abuse		–	–	–	x	x
Bullying		–	–	–	x	x
Daily use of electronic devices		–	x	–	x	x
Problematic social media use	Three items adapted from the Short Version of Internet Addiction Test (Müller et al., 2017)	–	x	–	x	x
Negative urgency	Short version of the UPPS‐P Impulsive Behavior Scale (SUPPS‐P; Cyders et al., 2014)	–	x	–	–	–
Positive urgency	SUPPS‐P (Cyders et al., 2014)	–	x	–	–	–
Resilience	Two items adapted from the Brief Resilience Scale (Smith et al., 2008)	–	x	–	–	–
Risk taking	One‐item general risk‐taking propensity measure (Dohmen et al., 2011)	–	x	–	–	–
Stress caused by work/studies		–	x	–	–	–
Factors related to school and studying		–	–	–	x	x
Neuroticism	Four items from the 100 Nuances of Personality (100‐NP; Henry & Mõttus, 2022)	–	–	x	–	–
Extraversion	Four items from the 100‐NP (Henry & Mõttus, 2022)	–	–	x	–	–
Openness to experience	Five items from the 100‐NP (Henry & Mõttus, 2022)	–	–	x	–	–
Agreeableness	Five items from the 100‐NP (Henry & Mõttus, 2022)	–	–	x	–	–
Conscientiousness	Four items from the 100‐NP (Henry & Mõttus, 2022)	–	–	x	–	–
D. *COVID‐19‐related stressors*						
Frontline worker status		x	x	x	–	–
Tested for COVID‐19		x	–	–	x	x
Diagnosed with COVID‐19		x	x	x	x	x
Severity of COVID‐19 symptoms		–	–	x	–	x
Perceived prejudiced attitudes due to COVID‐19		x	x	x	–	–
Vaccinated/plans to vaccinate against COVID‐19^d^		–	x	x	–	x
Changes in employment status		x	x	x	–	–
Perceived changes in mental health		x	–	x	–	–
Number of times spent in self‐isolation		–	x	–	x	–
Adherence to the rules of self‐isolation		–	x	–	x	–
Stress caused by measures to prevent the spread of COVID‐19^c^, ^d^		x	x	x	x	x
Personal measures to prevent the spread of COVID‐19		x	x	x	x	x
Best mood‐improving activities during the COVID‐19 crisis		–	x	–	–	–
E. *Mental health support during the crisis*						
Extent to which measures used to deal with the COVID‐19 crisis helped^c^, ^d^		x	–	x	x	x
Obstacles in using available help		x	–	–	–	–
Forms of help most lacking during the state of emergency in spring 2020		x	–	–	–	–
Forms of help most lacking at present		–	–	x	–	x
Forms of mental health support that could be more accessible to people during a crisis		x	–	–	–	–
F. *Anchoring vignettes*						
Anchoring vignettes for general health^b^, ^e^	Four items by Grol‐Prokopczyk (2018)	–	–	x	–	–
Anchoring vignettes for depression^b^, ^e^	Four items by King (n.d.)	–	–	x	–	–

Abbreviations: W1, wave 1; W2, wave 2; W3, wave 3.

^a^ In wave 2 web survey, these items were displayed to wave 1 non‐respondents only.

^b^ Items used in web survey only.

^c^ Different items for adults and minors.

^d^ Different sets of items in different survey waves.

^e^ Items displayed to one third of randomly selected subsamples.

Wave 2 and 3 questionnaires for minors were mostly adapted from the questionnaires for adults. The following changes were made: (a) considering the anonymity of responses, participant's age was asked instead of birth date; (b) irrelevant questions for this age group were omitted (e.g. occupation, income); (c) wording of some of the items was simplified; (d) questions about attitudes toward school and studying were added. Questionnaires for minors are presented in Additional files 4 and 5.

#### 2.3.3 Data processing

Completed postal questionnaires were entered manually using data entry form on the LimeSurvey platform. A random selection of 100 questionnaires from each wave was double entered for quality control. An error rate of < 0.5% was considered acceptable.

Given the mixed‐mode design and multiple contacts, dataset was screened for duplicate responses. If the same survey ID number appeared more than once in the dataset, the entry with more items answered was kept. Cases with invalid survey numbers were excluded. In both postal and web survey data (excepting anonymous sample of minors), answers on gender and date of birth were verified against the Population Register data. Inconsistencies were checked for possible data entry errors (postal surveys) and cases with remaining inconsistencies were excluded from the data. A questionnaire was considered completed if more than 50% of items on mental health (section B in the questionnaire; excluding free‐text questions and conditional items) were answered.

The EMHS uses different sets of post‐stratification weights to adjust for the potential response bias in the data. Given that the initial study sample consisted of 17 regional subsamples (15 counties and 2 cities), each representative for respective population's sex and age distribution, the calculated weights are meant to adjust the responses to enable analysis at both regional and total population level. Simple proportional weighting was used with coefficients found by dividing the proportion of each 5‐year age group for men and women in the population by the same proportion in the study data. Weights were calculated separately for each survey wave. Data were pre‐processed using statistical software R (R Core Team, 2021).

### 2.4 Registry study

Registry study, based on the survey sample (*n* = 20,000), aimed to complement and validate the survey data and to estimate the non‐response bias. Individual level data were linked from six administrative databases/registries on demographic, socioeconomic, health, and environmental characteristics:

1. Population Register data cover the date of birth, sex, legal marital status, citizenship, place of residence and self‐reported ethnicity, native language, and highest completed educational level.
2. Education Information System database is document based and includes data from 1998 as at earliest (database was established in 2004). The data about the level of completed education and of ongoing studies is used to replace/complete self‐reported Population Register data on education.
3. Social Insurance Board data cover monthly received social benefits and allowances from government and/or local authorities, social tax payments (used as a proxy for employment‐based income) and data on disabilities in January 1, 2019to March 1, 2021.
4. Unemployment Insurance Fund data include unemployment periods, the degree and the underlying ICD‐10 diagnosis code of incapacity for work, received allowances and benefits with exact dates in January 1, 2019 to March 1, 2021.
5. Health Insurance Fund data from medical services invoices cover ICD‐10 diagnosis codes, dates, and details of treatments/services received. Data on prescription medicines include drug name(s) and associated ICD‐10 diagnosis, date of prescription/purchase, quantity of packages, and costs. Data on health insurance coverage were also acquired. All data refer to the period January 1, 2016 to March 1, 2021.
6. Topographic database, administered by the Estonian Land Board was used to get environmental/contextual data (e.g., population density, type of living environment, types and total area of buildings, length and type of roads, average height of vegetation, distance from water bodies, presence of social infrastructure, and so on) to study the associations between person's daily living environment and mental health. All contextual variables were calculated on three spatial scales: the area of 100, 500, and 1000 m radius of the place of residence of each person in sample, respectively.

To obtain necessary data, inquiries were first made to the databases/registries (except Topographic database) about the possible data content and regulations of safe data transfer. After approving the formal request, the personal identification numbers together with personal study numbers were forwarded to the registries by authorized data analyst. The requested data were returned without personal identification number. All data exchanges with the registries were encrypted. To acquire environmental/contextual data, participants’ home addresses were converted into geographical coordinates using the Estonian Address Data System and were then linked to Topographic Database, both available as open data. Personal study numbers were then used to link registry and survey data with contextual information available from open topographical databases.

### 2.5 Validation study

To validate self‐assessed indicators, a subsample of wave 1 survey respondents was invited to participate in the validation study carried out simultaneously with the wave 2 survey. Only web respondents with a valid email address were considered for validation study; because the study was carried out in Estonian, an additional inclusion criterion was good command of the Estonian based on either registry data or wave 1 survey. By these criteria, 3698 participants were invited to the validation study including momentary assessments, emotions, and emotion regulation (five daily assessments during seven consecutive days), and daily reports of bedtime, alcohol consumption, physical activity, and self‐reported health. Of them, a subsample of 1000 participants living in selected 6 areas close to the study center were also invited to wear an activity monitor for the assessment of physical activity and sleep, and to donate a saliva sample for cortisol assessment. Further details on the validation study are given in Additional File 6.

### 2.6 Data management

Data are stored and managed at the NIHD. Personal data were used solely for contacting participants and making enquiries in national databases. Thereafter data were pseudonymized and personal study number was used to link survey data with data received from registries. The password‐protected key file linking participants’ personal data to their personal study number is stored separate from other study data on a limited‐access NIHD server for further research in accordance with the ethical committee approval. The key file will be preserved until December 31, 2030 and will be deleted thereafter. The anonymous data will be preserved indefinitely and will be available on motivated request.

### 2.7 Ethics

The study protocol and its amendments were approved by the Research Ethics Committee of the NIHD, Estonia (decisions no. 554 of November 27, 2020, no. 612 of January 21, 2021, no. 700 of March 31, 2021, no. 817 of August 6, 2021, and no. 931 of October 28, 2021). Written informed consent for participation was not required in accordance with national legislation and as approved by the Ethics Committee. Participants were informed that responding to the questionnaire was considered consenting to participate in the survey and granting permission for enquiries to be made in national databases. Consent was requested separately for each study wave and for the validation study.

## 3 RESULTS

Table 2 presents the sex and age distributions for eligible adult samples and response rates by survey wave. In wave 1, a total of 5636 persons provided complete responses, 3751 in wave 2 and 4744 in wave 3, yielding crude response rates of 30.1%, 21.0%, and 27.3%, respectively. Adjusted response rates, calculated by excluding those unavailable for the survey due to death, emigration, or deficient contact info (*n* = 306 in wave 1, *n* = 118 in wave 2, and *n* = 196 in wave 3) were slightly higher, 30.6%, 21.1%, and 27.6% in corresponding waves. In all waves, women were more likely to respond than men. Survey response differed also by age: older age groups (60 years and older) consistently exhibited higher response rates than younger age groups. Response rates were particularly low among 18−29‐year olds. Although the minors were recruited to the study anonymously and their responses cannot be linked to the sample data, the overall response rates in the 15−17 age group were 20.7% (*n* = 253) and 20.1% (*n* = 250), respectively, in waves 2 and 3. In total, 725 individuals declined their participation in the study and their data were removed from final analytical sample. Different sets of weights were calculated to adjust the responses and enable analysis at both national and regional levels. The weights are added to the dataset for the analytical sample.

**TABLE 2:** Sex and age distribution of the adult samples, response rates, and the prevalence of depression symptoms by survey wave

	Eligible sample	Respondents	Crude response rate	Adjusted response rate	Prevalence of symptoms of depression	
	*n*	*n*	%	%	%	95% CI
**Wave 1**						
Total	18,718	5636	30.1	30.6	27.6	25.4−29.8
Sex						
Male	9297	2257	24.3	24.7	23.9	20.8−27.3
Female	9421	3379	35.9	36.4	30.8	27.9−33.9
Age group^a^						
18−29	3339	803	24.0	24.3	48.2	42.0−54.4
30−44	4458	1108	24.9	25.1	26.8	22.7−31.3
45−59	4394	1417	32.2	32.7	21.7	18.1−25.8
60−74	3632	1340	36.9	37.5	20.5	16.4−25.3
75+	2895	968	33.4	34.7	25.6	19.7−32.5
**Wave 2**						
Total	17,874	3751	21.0	21.1	25.1	22.4−28.1
Sex						
Male	8963	1426	15.9	16.0	23.1	18.7−28.1
Female	8911	2325	26.1	26.2	26.9	23.6−30.5
Age group^a^						
18−29	3286	383	11.7	11.7	47.8	38.2−57.6
30−44	4394	647	14.7	14.8	25.4	20.3−31.3
45−59	4303	926	21.5	21.7	18.3	14.2−23.3
60−74	3448	1,026	29.8	30.0	17.7	13.4−23.0
75+	2443	769	31.5	31.8	20.5	14.2−28.7
**Wave 3**						
Total	17,362	4744	27.3	27.6	25.6	23.2−28.2
Sex						
Male	8729	1837	21.0	21.3	22.0	18.3−26.1
Female	8633	2907	33.7	34.0	28.8	25.7−32.1
Age group^a^						
18−29	3271	525	16.1	16.1	38.6	30.6−47.2
30−44	4341	873	20.1	20.3	29.1	24.1−34.6
45−59	4214	1264	30.0	30.4	19.9	16.2−24.2
60−74	3318	1275	38.4	38.9	19.1	15.0−23.9
75+	2218	807	36.4	37.0	22.6	17.4−28.8

^a^ Based on the age at the time of initial sampling.

^b^ Number of respondents divided by the eligible sample size excluding those who had died, emigrated, or had deficient contact info.

^c^ Weighted data.

The comprehensive overview of the prevalence of mental health problems in Estonian population remains out of the scope of this paper. Nevertheless, such data, even if limited to selected indicators may be highly informative to shed light on the general reliability and usefulness of data collected in EMHS, whose design and methods have been thoroughly described in this paper. Therefore, we present here the weighted prevalence estimates for depression, one of the most common mental disorders, as measured with the EST‐Q2 (Aluoja et al., 1999) (Table 2). Based on the previously established cut‐offs (Ööpik et al., 2006), about a quarter of respondents were at increased risk for depression (27.6%, 25.1%, and 25.6%, respectively, in waves 1, 2, and 3). Throughout the survey, women had higher prevalence of depression symptoms than men, the respective percentages in waves 1, 2, and 3 were 30.8%, 26.9%, and 28.8% among women, and 23.9%, 23.1%, and 22.0% among men. In all waves, young adults aged 18−29 years were at the highest risk for depression (48.2%, 47.8%, and 38.6% in waves 1, 2, and 3, respectively) compared to older age groups.

## 4 DISCUSSION

Triggered by the lack of nationwide up‐to‐date mental health data, the EMHS was launched to provide a comprehensive overview of the mental health and well‐being of the Estonian population. The need for such data became increasingly apparent upon the arrival of the COVID‐19 pandemic in 2020, as the first indications of its detrimental impact on population mental health became evident (COVID‐19 Mental Disorders Collaborators, 2021; Fountoulakis et al., 2021; OECD, 2021; Reile et al., 2021; Wang et al., 2020). In line with the cited studies, the results of the EMHS presented in this paper indicated high prevalence of symptoms of depression and revealed a clear rise compared with pre‐pandemic Estonian Health Interview Survey 2019 (Eesti rahvastiku vaimse tervise uuringu konsortsium, 2022). The rate of mental health problems was particularly high among young adults and women, also corroborating the findings from other studies (COVID‐19 Mental Disorders Collaborators, 2021; OECD, 2021).

It should be noted, however, that the presence of significant depression symptoms does not necessarily mean fulfilment of diagnostic criteria for depression: estimates based on screening instruments are typically higher than those based on full diagnostic interview. Nevertheless, knowing the accuracy of the instrument, it may be possible to find a more realistic estimate. Such information is available for the EST‐Q2 depression scale: in a reasonably large study with diagnostic interview as a criterion, the positive predictive value of the scale was found to be 0.44 and the negative predictive value was 0.96 (Ööpik et al., 2006). Using these values, one can predict that 44% of the participants who screened positive for depression (27.6% of the sample in wave 1) and 4% (the probability of a case being missed in screening) of the remaining sample (72.4%) would fulfil the diagnostic criteria, amounting to an overall prevalence estimate of approximately 15% ([0.276 × 0.44 + 0.724 × 0.04] × 100%). The corresponding estimates for waves 2 and 3 would be 14%.

The EMHS has several strengths compared to a number of previous studies. The EMHS is a large‐scale population‐based study covering a wide spectrum of mental health disorders and related issues that enables in‐depth analysis and policy recommendations on population level. The study is extremely timely in addressing the mental health problems associated with the COVID‐19 pandemic and its wider impact on the psychosocial environment. Longitudinal approach with survey waves at three time points in 2021–2022 is particularly useful to assess the development and/or persistence of mental health disorders during the pandemic. Linking survey data with registry‐based data on individual level allows to compare self‐reported symptoms of mental health disorders with information in electronic databases (e.g., diagnoses of mental disorders) and thus, to assess the consistency of the two approaches. Finally, the use of regionally stratified sampling allows to study (previously unavailable) regional variance in mental health indicators. As such, the EMHS has a strong potential to make a substantial contribution to the field of public mental health at both academic and applied levels.

The EMHS also has some limitations. One of the most serious challenges for the study was to recruit sufficient number of participants to allow reliable conclusions and guarantee the generalizability of the results. Low response rate in population‐based surveys has become increasingly common (Galea & Tracy, 2007) with online surveys on average yielding substantially lower response rates compared with other survey modes (Wu et al., 2022). The EMHS is not an exception as the overall adjusted response rates varied from 30.6% in wave 1 to 27.6% in wave 3. Low response rate itself does not necessarily mean biased results if responders and non‐responders do not differ systematically with respect to the outcome variable of interest. The good compliance with other recent studies regarding the prevalence of symptoms of depression allow us to believe that the results reflect the true pattern of mental health in the population. Another important limitation is the shortage of validated screening tools to detect mental disorders available in Estonian. The depression subscale of the EST‐Q2 is so far the only screening scale in Estonian that has been calibrated against clinical diagnosis (Ööpik et al., 2006). In the framework of the EMHS, several internationally well‐known measures (e.g., subscales from the DSM‐5 Screener) were adapted to Estonian, however, more research is needed to confirm their validity and calibrate them against clinical diagnoses. Finally, because of the legal restrictions and study time frame we were only able to include the minors, aged 15−17 years, in two of the three waves of the study and conduct the data collection anonymously.

## 5 CONCLUSIONS

The data collected in the EMHS serves as a solid foundation for planning mental health policies both generally and during the crisis. Given a wide spectrum of mental health disorders covered, the EMHS offers the rich baseline to monitor the changes and build up a full‐scale mental health surveillance system in Estonia. No less importantly, the EMHS allows valuable new insights into the determinants of mental health and well‐being and enables to elucidate some mechanisms for developing mental disorders, particularly during the crisis. Such information is particularly valuable given the scarcity of mental health studies in the Eastern European region.

## CONFLICT OF INTEREST STATEMENT

The authors declare no conflict of interest.

### PEER REVIEW

The peer review history for this article is available at https://publons.com/publon/10.1002/brb3.3106

## Supporting information

Additional file 1. Wave 1 questionnaire for adults (PDF)

Additional file 2. Wave 2 questionnaire for adults PDF)

Additional file 3. Wave 3 questionnaire for adults (PDF)

Additional file 4. Wave 2 questionnaire for minors (PDF)

Additional file 5. Wave 3 questionnaire for minors (PDF)

Additional file 6. Methods of the validation study (PDF)

## Data Availability

Data collected in the study are available from the authors upon request.
