# Supplementary material for: Estonian National Mental Health Study: Design and methods for a registry‐linked longitudinal survey
Source: Brain Behav. 2023 Jun 5;13(8):e3106. doi: 10.1002/brb3.3106 (PMC10454261; doi:10.1002/brb3.3106)
Supplement: Supplementary file 1 — Additional file 1. Wave 1 questionnaire for adults (PDF) [file BRB3-13-e3106-s006.pdf]

ESTONIAN NATIONAL MENTAL HEALTH STUDY: WAVE 1 QUESTIONNAIRE FOR ADULTS

Thank you for agreeing to participate in the Estonian National Mental Health Study. The following questionnaire is about various aspects of your well-being. **To choose an answer option, circle the number next to the appropriate option or write it in the designated space. Every response is very important to us.** We assure that all your answers are treated with full confidentiality.

First, we will ask you some questions about your general life situation.

A1. Sex 1 Male 2 Female

A2. Date of birth |__|__| |__|__| |__|__|__|__|
Day Month Year

A3. Ethnicity
1 Estonian
2 Russian
3 Other. Please specify: …………..……………...……………….

A4. What is the main language you speak at home?
1 Estonian
2 Russian
3 Other. Please specify: ………………….……………………….…….….……...…...……

A5. What is your current marital status?
1 Single (never been married/cohabited)
2 Married or cohabiting
3 In a steady relationship but do not live together
4 Divorced/separated
5 Widowed

A6. What is the highest level of education you have completed?
1 Primary education (up to 6 years)
2 Basic education
3 Basic education with vocational training
4 Secondary education
5 Vocational secondary education
6 Higher vocational education
7 Undergraduate degree (Bachelor's)
8 Postgraduate degree (Master’s, PhD/candidate)

A7. Which of the following is the most accurate description of your current employment status? Select one primary status.

1 I am studying or doing an unpaid internship → *Proceed to question A8*

2 I am employed/working as a contractor → *Proceed to question A8*

3 I am an entrepreneur → *Proceed to question A8*

4 I am registered as unemployed

5 I am unemployed and not actively seeking employment

6 I am an old-age pensioner → *Proceed to question A8*

7 I have been declared incapacitated for work → *Proceed to question A8*

8 I am on parental leave → *Proceed to question A8*

9 I am a homemaker → *Proceed to question A8*

10 I am in military service → *Proceed to question A8*

11 I am the caregiver to a close relative → *Proceed to question A8*

12 Other. Please specify: …………………………………………………….…………………

A7a. How many months ago did you last work?

1 ...... months ago (Write the number of months if less than a year.)

2 More than 12 months ago

A8. What else do you do in addition to your primary activity? Select all applicable responses.

1 I do nothing else

2 I am studying or doing an unpaid internship

3 I am employed/working as a contractor

4 I am an entrepreneur

5 I am an old-age pensioner

6 I have been declared incapacitated for work

7 I am the caregiver to a close relative

8 Other. Please specify: …………………………..………………………………………...

A9. What is your current or most recent occupation? Select one primary occupation.

1 I have never worked

2 Legislator, senior official, manager (public official, director, executive, etc.)

3 Professional (engineer, physician, software developer, lecturer, teacher, researcher, creative professional, etc.)

4 Associate professional (technician, inspector, nurse, real estate agent, social worker, etc.)

5 Official, customer service representative (secretary, clerk, administrator, etc.)

6 Service or sales staff (guide, chef, cashier, babysitter, police officer, prison official, salesperson, janitor, etc.)

7 Skilled labourer in agriculture, fishery, forestry or hunting (gardener, breeder, fisher, farmer, etc.)

8 Skilled worker (miner, carpenter, electrician, printing worker, tailor, craftsman, etc.)

9 Device or machine operator (operator, assembler, bus driver, crane operator, etc.)

10 Unskilled worker (guard, cleaner, street vendor, agricultural worker, transport worker, etc.)

11 Professional soldier

A10. What is your average monthly net income for the last 12 months? (net income from all sources, including family allowance, pensions and rental income)

1 No income

2 Up to 450 euros

3 451–650 euros

4 651–850 euros

5 851–1100 euros

6 1101–1400 euros

7 1401–1700 euros

8 1701–2000 euros

9 2001–2500 euros

10 2501 euros or more

A11. What is the current financial situation of your household? A household is generally comprised of people living in the same dwelling who share food or a budget.

1 We have money to spare

2 We have enough money to get by

3 We are barely making ends meet

4 We do not have enough money to cover unavoidable costs (such as utility bills)

5 Cannot say

A12. How many people live in your household?

1 I live alone → *Proceed to question A14*

2 There are ...... people in addition to me

A12a. How many children live in your household? Include yourself, if applicable. Mark 0 if no children of the given age live in your household.

.... children under 7 years of age

…... children aged 7–17

…... children aged 18–19 studying in a general education school

A13. Who currently belong to your household? Mark everyone you live with.

1 Spouse/partner
2 One parent
3 Both parents
4 One or more in-laws
5 One or more grandparents
6 One or more siblings
7 One or more children (including adult children)
8 One or more grandchildren
9 Other. Please specify:

……………………..…………

A14. Does anyone in your household require constant care due to an illness or a medical condition? Select all applicable responses.

1 No one requires care
2 I require care myself
3 Someone else in the household requires care

A15. In your permanent residence, how many rooms are in use by your household? Do not count the kitchen and other ancillary premises.

…… rooms

A16. Where is your current residence?

1 Harju County, excluding Tallinn
2 Tallinn
3 Hiiu County
4 Ida-Viru County
5 Jõgeva County
6 Järva County
7 Lääne County
8 Lääne-Viru County
9 Põlva County
10 Pärnu County
11 Rapla County
12 Saare County
13 Tartu County, excluding Tartu
14 Tartu
15 Valga County
16 Viljandi County
17 Võru County
18 I do not reside in Estonia. Please specify your country of residence:

....

A17. What type of settlement do you currently reside in?

1 A settlement with a population of less than 1000 or in the countryside
2 A settlement with a population of 1000–10,000
3 A settlement with a population of more than 10,000

The following questions are about your well-being, how you feel and your mental health.

Please rate your satisfaction with the following aspects of your life:

		Very satisfied	Quite satisfied	Not particularly satisfied	Not satisfied at all	
**B1.**	Life in general	1	2	3	4	
**B2.**	Your financial situation	1	2	3	4	
**B3.**	Family relations	1	2	3	4	
**B4.**	Friendships	1	2	3	4	
**B5.**	Work	1	2	3	4	5<br>I do not work

B6. Have you ever been diagnosed with a mental disorder (such as depression, anxiety or alcoholism)?

1 No

2 Yes

3 Don't know

4 Prefer not to answer

For the following questions, please indicate the answer that best describes your behaviour and how you have felt <u>over the past six (6) months</u>.

		Never	Rarely	Some-times	Often	Very often
**B7.**	How often do you struggle to complete a task once the more exciting parts of it are completed?	1	2	3	4	5
**B8.**	How often do you struggle with tasks that require systematisation or organisation?	1	2	3	4	5
**B9.**	How often do you have problems with remembering meetings or responsibilities?	1	2	3	4	5
**B10.**	How often do you avoid or postpone activities or tasks that demand great mental effort?	1	2	3	4	5
**B11.**	How often do you start fidgeting or moving your arms/legs unintentionally if you have to sit in one place for a long time?	1	2	3	4	5
**B12.**	How often do you feel overactive and restless as if you have been ‘wound up’?	1	2	3	4	5

Carefully read the following list of problems and complaints that people sometimes experience. Please indicate how much each one has bothered you <u>during the last four (4) weeks</u>.

		Not at all	Rarely	Some-times	Often	Constantly
**B13.**	Sadness	1	2	3	4	5
**B14.**	Lack of interest in things	1	2	3	4	5
**B15.**	Feeling of worthlessness	1	2	3	4	5
**B16.**	Self-accusations	1	2	3	4	5
**B17.**	Recurrent thoughts of death or suicide	1	2	3	4	5
**B18.**	Feeling lonely	1	2	3	4	5
**B19.**	Hopelessness about the future	1	2	3	4	5
**B20.**	Inability to feel joy	1	2	3	4	5
**B21.**	Feeling easily irritated or annoyed	1	2	3	4	5
**B22.**	Feeling anxious or fearful	1	2	3	4	5
**B23.**	Feeling tense or unable to relax	1	2	3	4	5
**B24.**	Excessive worry about several things	1	2	3	4	5
**B25.**	Feeling so anxious or restless that it is hard to sit still	1	2	3	4	5
**B26.**	Being easily startled	1	2	3	4	5
**B27.**	Sudden panic attacks with palpitations, shortness of breath, faintness or other distressing bodily sensations	1	2	3	4	5
**B28.**	Fear of being away from home alone	1	2	3	4	5
**B29.**	Feeling afraid in public spaces or on the street	1	2	3	4	5
**B30.**	Fear of fainting in public	1	2	3	4	5
**B31.**	Fear of travelling by bus, tram, train or car	1	2	3	4	5
**B32.**	Fear of being the centre of attention	1	2	3	4	5
**B33.**	Fear of interacting with strangers	1	2	3	4	5

		Not at all	Rarely	Some-times	Often	Constantly
**B34.**	Fatigue or loss of energy	1	2	3	4	5
**B35.**	Diminished attention span or ability to concentrate	1	2	3	4	5
**B36.**	Resting does not restore strength	1	2	3	4	5
**B37.**	Being easily fatigued	1	2	3	4	5
**B38.**	Difficulty falling asleep	1	2	3	4	5
**B39.**	Restless or disturbed sleep	1	2	3	4	5
**B40.**	Waking up too early	1	2	3	4	5
**B41.**	Deliberate self-harm (such as intentionally cutting your skin or causing pain, hitting yourself)	1	2	3	4	5

How much (or how often) have the following problems or complaints bothered you <u>during the last four (4) weeks?</u>

		Not at all	Rarely	Some-times	Often	Constantly
**B42.**	Sleeping less than usual, but still have a lot of energy	1	2	3	4	5
**B43.**	Starting lots more projects than usual or doing more risky things than usual	1	2	3	4	5
**B44.**	Unexplained aches and pains (e.g., head, back, joints, abdomen, legs)	1	2	3	4	5
**B45.**	Feeling that your illnesses are not being taken seriously enough	1	2	3	4	5
**B46.**	Hearing things other people couldn’t hear, such as voices even when no one was around	1	2	3	4	5
**B47.**	Feeling that someone could hear your thoughts, or that you could hear what another person was thinking	1	2	3	4	5

		Not at all	Rarely	Some-times	Often	Constantly
**B48.**	Problems with memory (e.g., learning new information) or with location (e.g., finding your way home)	1	2	3	4	5
**B49.**	Unpleasant thoughts, urges, or images that repeatedly enter your mind	1	2	3	4	5
**B50.**	Feeling driven to perform certain behaviors or mental acts over and over again	1	2	3	4	5
**B51.**	Feeling detached or distant from yourself, your body, your physical surroundings, or your memories	1	2	3	4	5

How much do the following statements apply to you? Please select the most applicable answer.

		Completely false	Mostly false	Neither true nor false	Mostly true	Completely true
**B52.**	Most of the time I feel lively and energetic.	1	2	3	4	5
**B53.**	Most of the time I feel attentive and alert.	1	2	3	4	5
**B54.**	I am hopeful and enthusiastic about the future.	1	2	3	4	5

B55. **Sometimes things happen to people that are particularly frightening or traumatic.** Such events can include natural disasters and other catastrophes, wars, serious accidents and fires, a serious illness, being placed under intensive care, sexual or physical assault or abuse, witnessing a murder, suicide or injuries and the sudden death of someone close. **Have you ever experienced such events?**

1 No, never → *Proceed to question B56*

2 Yes, more than a month ago

3 Yes, less than a month ago

Below is a list of problems and complaints that people sometimes have in response to stressful experiences. Please indicate how much each problem has bothered you <u>during the last four (4) weeks.</u>

		Not at all	Rarely	Sometimes	Often	Constantly
**B55a.**	Repeated, disturbing memories, thoughts or images of a stressful experience	1	2	3	4	5
**B55b.**	Feeling very upset when something reminded you of a stressful experience	1	2	3	4	5
**B55c.**	Avoiding activities or situations because they reminded you of a stressful experience	1	2	3	4	5
**B55d.**	Being watchful or easily startled	1	2	3	4	5

Next, we want to know the importance of food and eating in your life <u>over the past three (3) months</u>.

B56. Have you spent a considerable amount of time thinking about food and your weight?

1 No 2 Yes

B57. Have you considerably limited your diet <u>over the past three months</u>?

1 No 2 Yes

B58. Have you been binge eating (eating more than usual) <u>over the past three months</u>?

1 No → *Proceed to question B59* 2 Yes

B58a. During these binges, have you felt that you cannot control your eating?

1 No 2 Yes

B59. Have you deliberately made yourself vomit, used laxatives or appetite suppressants to control your weight <u>over the past three months</u>?

1 No 2 Yes

The following questions are about your behaviour <u>over the past 12 months</u>.

B60. Have you gambled in casinos or online, or bought lottery tickets <u>in the last 12 months</u>?

1 No → *Proceed to question C1* 2 Yes

B61a. Have you become restless, irritable or anxious when trying to stop/cut down your gambling?

1 No 2 Yes

B61b. Have you tried to keep your family or friends from knowing how much you have gambled?

1 No 2 Yes

B61c. Did you have such financial trouble as a result of your gambling that you had to get help with your living expenses from family, friends or welfare?

1 No 2 Yes

In the following section we will be asking about your general health and health behaviour.

C1. How tall are you? (without shoes) ……… cm

C2. How much do you weigh? (without clothes) If you are pregnant, note your weight prior to your pregnancy.

……… kg

C3. Do you have valid health insurance?

1 No 2 Yes

C4. How would you assess your current state of health?

1 Very good
2 Good
3 Average
4 Poor
5 Very poor

C5. Do you have any longstanding (chronic) illness or health problem?

1 No 2 Yes

C6. How concerned have you been about your health over the past six (6) months?

1 Not at all
2 A little
3 To some extent
4 A lot
5 Very much

Under each heading, please select the answer option that best describes your health today.

C7. Mobility:

1 I have no problems in walking about
2 I have some problems in walking about
3 I am confined to bed

C8. Self-care:

1 I have no problems with self-care
2 I have some problems washing or dressing myself
3 I am unable to wash or dress myself

C9. Usual activities (e.g. work, study, housework, family or leisure activities):

1 I have no problems with performing my usual activities

2 I have some problems with performing my usual activities

3 I am unable to perform my usual activities

C10. Pain / discomfort:

1 I have no pain or discomfort

2 I have moderate pain or discomfort

3 I have severe pain or discomfort

C11. Anxiety / depression:

1 I am not anxious or depressed

2 I am moderately anxious or depressed

3 I am extremely anxious or depressed

C12. <u>Over the past three (3) months</u>, how often in your leisure time have you been active (playing sports, doing gardening, high-speed cycling or brisk walking, etc.) for at least 30 min at a time so that you are slightly out of breath or sweating?

1 Never

2 Once a month or less

3 2–3 times per month

4 Once a week

5 2–3 times a week

6 4–6 times a week

7 Every day

C13. Have you smoked <u>in the past three (3) months</u>?

1 No → **C13a. Have you ever smoked?**

1 I have never smoked

2 I quit more than 6 months ago

3 I quit less than 6 months ago

2 Yes → **C13b. What characterises your smoking?** Select all applicable responses.

1 I smoke cigarettes/cigars/a pipe daily

2 I smoke e-cigarettes or other smoke-free products daily

3 I smoke cigarettes/cigars/a pipe occasionally

4 I smoke e-cigarettes or other smoke-free products occasionally

C14. How often have you had a drink containing alcohol <u>over the past three (3) months</u>?

1 Never → *Proceed to question C17*

2 Monthly or less

3 2–4 times per month

4 2–3 times per week

5 4 or more times per week

C15. How many units of alcohol did you usually consume at one time <u>in the past three (3) months</u>?

1 1–2

2 3–4

3 5–6

4 7–9

5 10+

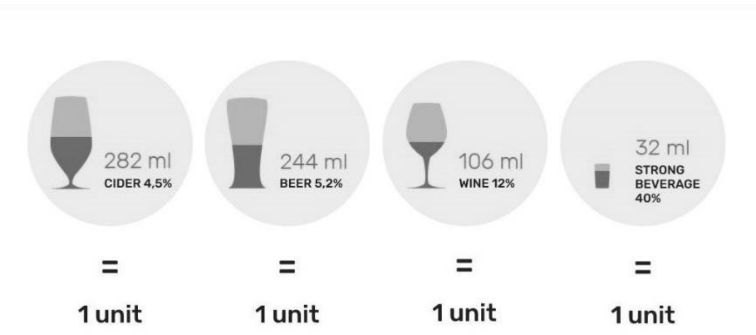

C16. How often have you had 6 or more units on a single occasion?

1 Never

2 Less than once a month

3 Once a month

4 Once a week

5 Every day or almost every day

C17. How often have you used narcotic substances <u>over the past three (3) months</u>? Narcotic substances include cannabis, cocaine or crack, 'party drugs' (such as ecstasy), hallucinogens (such as LSD), heroine, solvents or inhalants (such as glue) or methamphetamine (such as speed).

1 Never

2 Once a month or less

3 2–4 times a month

4 2–3 times a week

5 4 or more times a week

C18. How many hours a day have you slept <u>in the past three (3) months</u>? Include naps in your calculation.

…… hours and …… minutes on a weekday

…… hours and …… minutes at the weekend

The following questions are about your emotions and how you have dealt with them <u>over the past three (3) months</u>.

C19. How often have you recently felt that difficulties were piling up so high that you could not overcome them?

1 Very rarely

2 Quite rarely

3 Sometimes

4 Quite often

5 Very often

C20. How often have you recently felt that your work is emotionally demanding?

1 Very rarely

2 Quite rarely

3 Sometimes

4 Quite often

5 Very often

Please rate how often the following statements apply to you.

C21. When I am upset, I have difficulty controlling my behaviours.

1 Almost never
2 Sometimes
3 Often
4 Most of the time
5 Almost always

C22. When I feel low-spirited, I only think about my problems and I find it hard to focus on anything else.

1 Almost never
2 Sometimes
3 Often
4 Most of the time
5 Almost always

C23. When I am upset, I believe that there is nothing I can do to make myself feel better.

1 Almost never
2 Sometimes
3 Often
4 Most of the time
5 Almost always

When I am feeling negative emotions and want to feel better…

C24. … I think about what I can learn from the situation.

1 Almost never
2 Sometimes
3 Often
4 Most of the time
5 Almost always

C25. ... I remind myself that I can usually manage negative situations.

1 Almost never
2 Sometimes
3 Often
4 Most of the time
5 Almost always

C26. ... I reconsider how important this situation actually is to me.

1 Almost never
2 Sometimes
3 Often
4 Most of the time
5 Almost always

C27. ... I think about how negative situations are just a part of life.

1 Almost never
2 Sometimes
3 Often
4 Most of the time
5 Almost always

Next, we will ask you to respond to questions about your relationships with your family and those close to you. Please rate how much the following statements apply to you.

C28. I can rely on my loved ones for help if something bad happens to me.

1 Completely disagree
2 Tend to disagree
3 Tend to agree
4 Completely agree

C29. How often does emotional abuse (swearing or yelling at you, insulting, etc.) occur in your family?

1 Never
2 Very rarely
3 It has occurred occasionally
4 Quite often
5 Almost every day

C30. Have you ever experienced domestic abuse of an emotional, sexual or physical nature? Domestic abuse is any mental, physical or sexual abuse which occurs between people who are or have at some point been in an intimate relationship or legally bound, or who are related by blood.

1 Never
2 Yes, but not within the last 12 months
3 Yes, within the last 12 months
4 Don't wish to say

The following questions are about your everyday life and how you have been dealing with it <u>since the start of the coronavirus epidemic in spring 2020</u>.

D1. Do you face a heightened risk of contact with COVID-19 infected people due to your profession?

1 No
2 Yes, I have worked as a doctor, nurse or pharmacist
3 Yes, I have worked as a caregiver
4 Yes, I have worked as a police officer
5 Yes, I have been a service worker
6 Yes, I have worked as a public transport driver
7 Yes, I have worked as a teacher
8 Yes, other. Please specify: ………………………………………………………………………

D2. Have you been tested for coronavirus?

1 No → *Proceed to question D3*
2 Yes

D2a. Please select your reason(s) for getting tested. Select all applicable responses.

1 I developed symptoms characteristic of coronavirus
2 I was in contact with someone who was diagnosed with coronavirus
3 I was in contact with someone who had symptoms characteristic of coronavirus

4 I was in a large crowd in a public space/at an event

5 I was travelling in a high-risk area

6 Just in case

7 I participated in a coronavirus study

8 Other. Please specify: ……………………..……………………………………………...…

D2b. Have you been diagnosed with coronavirus?

1 No, I have not been diagnosed with coronavirus

2 Yes, I have been diagnosed with coronavirus

D2c. Do you feel you have been subject to unfavourable, negative or prejudiced attitude due to your possible contact with coronavirus?

1 No 2 Yes

D3. Has your employment status changed in relation to the coronavirus crisis (since spring 2020)?

1 It has not changed

2 Yes, I am working more than before

3 Yes, I am working less than before

4 Yes, I decided to remain at home with the children

5 Yes, I decided to retire

6 Yes, I was laid off or became unemployed

7 Other. Please specify: ……………………………………………………….………………

D4. Have you noticed any changes in your mental health since the start of the coronavirus crisis compared to the time before the crisis?

1 My mental health has declined

2 My mental health has remained unchanged

3 My mental health has improved

4 I don’t know

Please rate how much the following measures to prevent the spread of coronavirus (in use <u>as of spring 2020</u>) and the characteristics of the state of emergency have caused you stress.

		Not applicable	Caused no stress	Caused some stress	Caused significant stress
**D5.**	Restrictions on shopping centres	1	2	3	4
**D6.**	Distance learning in primary school	1	2	3	4
**D7.**	Distance learning in basic and secondary schools and universities	1	2	3	4

		Not applicable	Caused no stress	Caused some stress	Caused significant stress
**D8.**	Limited access to childcare / kindergartens	1	2	3	4
**D9.**	Reorganisation of work, e.g. remote working	1	2	3	4
**D10.**	Restrictions on entertainment establishments (such as theatres, cinemas, museums, exhibitions, concerts, cafés and restaurants)	1	2	3	4
**D11.**	Restrictions on organising events, including official and family events (such as graduations, birthday parties, weddings and funerals)	1	2	3	4
**D12.**	Restrictions on sports facilities (such as fitness clubs, stadiums, outdoor gyms and playgrounds)	1	2	3	4
**D13.**	Restrictions on visiting churches or other religious establishments	1	2	3	4
**D14.**	The 2+2 rule in public spaces	1	2	3	4
**D15.**	The compulsory isolation of those infected and their contacts	1	2	3	4
**D16.**	The closing of borders and introduction of travel restrictions	1	2	3	4
**D17.**	Self-isolation when travelling from abroad	1	2	3	4
**D18.**	Restrictions on alcohol sales in bars/restaurants	1	2	3	4
**D19.**	Temporary interruptions to planned medical treatment	1	2	3	4
**D20.**	Reduced access to social services (family care, personal care or home care services)	1	2	3	4
**D21.**	Visiting restrictions (including friends, elderly family members and loved ones in hospitals and nursing homes)	1	2	3	4
**D22.**	Economic or labour market uncertainty	1	2	3	4
**D23.**	Limited opportunities for social interaction	1	2	3	4
**D24.**	Recommended / required mask wearing in public spaces	1	2	3	4

D25. With all things considered, how much stress did the state of emergency in spring 2020 cause for you?

1 Not at all
2 A little
3 Somewhat
4 A lot
5 Very much

D26. How stressed do you currently feel due to the coronavirus crisis?

1 Not at all
2 A little
3 Somewhat
4 A lot
5 Very much

D27. What have you done in the past four (4) weeks to prevent yourself or others from becoming infected with coronavirus? Select all of the measures you have taken.

1 Regularly washing and disinfecting your hands
2 Covering your mouth and nose when coughing or sneezing
3 Wearing a mask or a visor
4 Keeping a safe distance from others
5 Avoiding events and gatherings
6 Avoiding shopping centres and grocery stores
7 Staying home at any sign of illness
8 Getting tested for coronavirus
9 Getting vaccinated against coronavirus
10 Avoiding public transport
11 Avoiding indoor public spaces
12 Staying at home
13 None of the above
14 Other. Please specify: ……………………………………………………….……………

To what extent have the following measures helped you deal with the coronavirus crisis since spring 2020?

		**Never used them**	**Did not help**	**Helped somewhat**	**Helped significantly**
**E1.**	Watching and listening to useful television and radio broadcasts or participating in webinars	1	2	3	4
**E2.**	Looking up additional information about my concerns online	1	2	3	4

		Never used them	Did not help	Helped somewhat	Helped significantly
**E3.**	Phoning helplines (such as the 1227 crisis helpline and the mental health helpline)	1	2	3	4
**E4.**	Seeing my primary care doctor (GP)	1	2	3	4
**E5.**	Using mental health web or phone apps	1	2	3	4
**E6.**	Seeing a psychiatrist, psychologist or psychotherapist	1	2	3	4
**E7.**	Talking to a priest or clergyman	1	2	3	4
**E8.**	Using different social services (such as personal care or home care to deliver medication and groceries or support at home)	1	2	3	4
**E9.**	Other. Please specify: ……………………...………………………………	1	2	3	4

E10. What prevented you from using available help? Select all applicable responses.

1 I did not feel the need for help/no obstacles

2 The problems resolved themselves or I tried to get by without help

3 I had no time to search for or use help

4 I did not know who to turn to for help

5 Planned treatment in medical and other mental health establishments was limited

6 The waiting lists for appointments in medical and other mental health establishments were too long

7 The services were too expensive

8 Family members or loved ones were against it

9 I was hesitant to seek help because I was afraid of being judged

10 I did not believe that anything would help

11 Other. Please specify: …………………………………………………………….………………

E11. What kind of help do you feel was most lacking during the state of emergency in spring 2020? Take into account both your own help needs as well as those of your loved ones. Select all applicable responses.

1 Health care

2 Mental health services (psychological or psychiatric help, and counselling)

3 Emotional support (including empathy and talking about your feelings)

4 Economic/financial aid

5 Childcare

6 Help in carrying out distance learning for my child/children

7 Personal care for myself (such as help dressing, washing myself, etc.)

8 Home care services for myself (such as food and medicine deliveries, help with cleaning and cooking)

9 Care services for loved ones

10 Personal care or home care for loved ones

11 Other services (such as beauticians, hairdressers and physiotherapy)

12 Other. Please specify: ……………………………………………………………...…….

13 I did not feel that anything was lacking

E12. Which of the following forms of mental health support (services) could be more accessible to people in need during a crisis? Select all applicable responses.

1 Primary care doctors (GPs)

2 Psychiatrist's consultations

3 Psychologist's or counsellor's consultations (including psychotherapy, counselling and family therapy)

4 Online or phone consultations with doctors, [mental health] nurses or counsellors

5 Crisis helplines

6 Victim support services

7 Pastoral care

8 Mental health websites listing help measures

9 Other. Please specify: ……………………………………………………………...…….

10 None of the above

Today's date [a] |__|__| |__|__| 2021
Day Month

You have now reached the end of the questionnaire. Please make sure that you have answered all the questions.

Thank you very much for taking the time to complete the questionnaire!

Do you wish to be entered in the gift voucher prize draw? 1 No 2 Yes

If you have any additional information that you would like to share with us, please do so in the space below.

[a] Only in postal survey
