# Supplementary material for: Estonian National Mental Health Study: Design and methods for a registry‐linked longitudinal survey
Source: Brain Behav. 2023 Jun 5;13(8):e3106. doi: 10.1002/brb3.3106 (PMC10454261; doi:10.1002/brb3.3106)
Supplement: Supplementary file 2 — Additional file 2. Wave 2 questionnaire for adults PDF) [file BRB3-13-e3106-s004.pdf]

ESTONIAN NATIONAL MENTAL HEALTH STUDY: WAVE 2 QUESTIONNAIRE FOR ADULTS

Thank you for agreeing to participate in the second wave of the Estonian National Mental Health Study. The following questionnaire is about various aspects of your well-being. **To choose an answer option, circle the number next to the appropriate option or write it in the designated space. Every response is very important to us.** We assure that all your answers are treated with full confidentiality.

First, we will ask you some questions about your general life situation.

A1. Sex 1 Male 2 Female

A2. Date of birth |__|__| |__|__| |__|__|__|__|

Day Month Year

A3.[a] What is your current marital status?

1 Single (never been married/cohabited)

2 Married or cohabiting

3 In a steady relationship but do not live together

4 Divorced/separated

5 Widowed

A3a.[ab] What is the highest level of education you have completed?

1 Primary education (up to 6 years)

2 Basic education

3 Basic education with vocational training

4 Secondary education

5 Vocational secondary education

6 Higher vocational education

7 Undergraduate degree (Bachelor's)

8 Postgraduate degree (Master’s, PhD/candidate)

A4.[a] Which of the following is the most accurate description of your current employment status? Select one primary status.

1 I am studying or doing an unpaid internship → *Proceed to question A6*

2 I am employed/working as a contractor → *Proceed to question A6*

3 I am an entrepreneur → *Proceed to question A6*

4 I am registered as unemployed

5 I am unemployed and not actively seeking employment

6 I am an old-age pensioner → *Proceed to question A6*

7 I have been declared incapacitated for work → *Proceed to question A6*

8 I am on parental leave → *Proceed to question A6*

9 I am a home-maker → *Proceed to question A6*

10 I am in military service → *Proceed to question A6*

11 I am the caregiver to a close relative → *Proceed to question A6*

12 Other. Please specify: ………………….…………………………………………………

A5.[a] How many months ago did you last work?

1 ...... months ago (Write the number of months if less than a year.)

2 More than 12 months ago

A6.[a] What else do you do in addition to your primary activity? Select all applicable responses.

1 I do nothing else

2 I am studying or doing an unpaid internship

3 I am employed/working as a contractor

4 I am an entrepreneur

5 I am an old-age pensioner

6 I have been declared incapacitated for work

7 I am the caregiver to a close relative

8 Other. Please specify: …………………………..………………………………………...

A7.[a] What is your current or most recent occupation? Select one primary occupation.

1 I have never worked

2 Legislator, senior official, manager (public official, director, executive, etc.)

3 Professional (engineer, physician, software developer, lecturer, teacher, researcher, creative professional, etc.)

4 Associate professional (technician, inspector, nurse, real estate agent, social worker, etc.)

5 Official, customer service representative (secretary, clerk, administrator, etc.)

6 Service or sales staff (guide, chef, cashier, babysitter, police officer, prison official, salesperson, janitor, etc.)

7 Skilled labourer in agriculture, fishery, forestry or hunting (gardener, breeder, fisher, farmer, etc.)

8 Skilled worker (miner, carpenter, electrician, printing worker, tailor, craftsman, etc.)

9 Device or machine operator (operator, assembler, bus driver, crane operator, etc.)

10 Unskilled worker (guard, cleaner, street vendor, agricultural worker, transport worker, etc.)

11 Professional soldier

A8.[a] What is your average monthly net income for the last 12 months? (net income from all sources, including family allowance, pensions and rental income)

1 No income
2 Up to 450 euros
3 451–650 euros
4 651–850 euros
5 851–1100 euros
6 1101–1400 euros
7 1401–1700 euros
8 1701–2000 euros
9 2001–2500 euros
10 2501 euros or more

A9.[a] What is the current financial situation of your household? A household is generally comprised of people living in the same dwelling who share food or a budget.

1 We have money to spare
2 We have enough money to get by
3 We are barely making ends meet
4 We do not have enough money to cover unavoidable costs (such as utility bills)
5 Cannot say

A10.[a] How many people live in your household?

1 I live alone → *Proceed to question A12*
2 There are ...... people in addition to me

A10a.[a] How many children live in your household? Include yourself, if applicable. Mark 0 if no children of the given age live in your household.

.... children under 7 years of age
…... children aged 7–17
…... children aged 18–19 studying in a general education school

A11.[a] Who currently belong to your household? Mark everyone you live with.

1 Spouse/partner
2 One parent
3 Both parents
4 One or more in-laws
5 One or more grandparents
6 One or more siblings
7 One or more children (including adult children)
8 One or more grandchildren
9 Other. Please specify: ……………………..…………

A12.[a] Does anyone in your household require constant care due to an illness or a medical condition? Select all applicable responses.

1 No one requires care
2 I require care myself
3 Someone else in the household requires care

A13.[a] **In your permanent residence, how many rooms are in use by your household?** Do not count the kitchen and other ancillary premises.

…… rooms

A14.[a] **Where is your current residence?**

1 Harju County, excluding Tallinn
2 Tallinn
3 Hiiu County
4 Ida-Viru County
5 Jõgeva County
6 Järva County
7 Lääne County
8 Lääne-Viru County
9 Põlva County
10 Pärnu County
11 Rapla County
12 Saare County
13 Tartu County, excluding Tartu
14 Tartu
15 Valga County
16 Viljandi County
17 Võru County
18 I do not reside in Estonia. Please specify your country of residence:

....

A15.[a] **What type of settlement do you currently reside in?**

1 A settlement with a population of less than 1000 or in the countryside
2 A settlement with a population of 1000–10,000
3 A settlement with a population of more than 10,000

A16. Do you live at your officially registered address?

1 No
2 Yes

A17. Describe the neighbourhood where you spend a significant portion of your time. Select all applicable responses.

1 Apartment building area at the edge of the city
2 Inner-city apartment building area
3 Low-density or mostly single-family housing area
4 City centre
5 Industrial area
6 Agricultural environment (e.g. fields, farms)
7 Green environment (e.g. park, forest, waterfront)
8 Vicinity of military or other defence-related facilities

Please rate to what extent you are disturbed by the following factors in your daily environment?

		Not at all	A little	To some extent	A lot	Very much
**A18.**	Noise (e.g., from industry, transport)	1	2	3	4	5
**A19.**	Noise (e.g., from human activity)	1	2	3	4	5
**A20.**	Street lights are too bright	1	2	3	4	5
**A21.**	Street lights are too dim	1	2	3	4	5
**A22.**	Air pollution	1	2	3	4	5
**A23.**	Smell	1	2	3	4	5
**A24.**	View	1	2	3	4	5
**A25.**	Lack of greenery	1	2	3	4	5

A26. Do you have any pets? Select all applicable responses.

1 No

2 Cat(s)

3 Dog(s)

4 Fish

5 Rodents (e.g. rabbit, hamster, guinea pig, rat)

6 Other. Please specify: …………………………………………….………….……………...

The following questions are about your well-being, how you feel and your mental health.

Please rate your satisfaction with the following aspects of your life:

		Very satisfied	Quite satisfied	Not particularly satisfied	Not satisfied at all	
**B1.**	Life in general	1	2	3	4	
**B2.**	Your financial situation	1	2	3	4	
**B3.**	Family relations	1	2	3	4	
**B4.**	Friendships	1	2	3	4	
**B5.**	Work	1	2	3	4	5<br>I do not work

B6. Have you been diagnosed with a mental disorder (such as depression, anxiety or alcoholism) <u>over the past three (3) months</u>?

1 No

2 Yes

3 Don't know

4 Prefer not to answer

Carefully read the following list of problems and complaints that people sometimes experience. Please indicate how much each one has bothered you <u>during the last four (4) weeks</u>.

		Not at all	Rarely	Some-times	Often	Constantly
**B7.**	Sadness	1	2	3	4	5
**B8.**	Lack of interest in things	1	2	3	4	5
**B9.**	Feeling of worthlessness	1	2	3	4	5
**B10.**	Self-accusations	1	2	3	4	5
**B11.**	Recurrent thoughts of death or suicide	1	2	3	4	5
**B12.**	Feeling lonely	1	2	3	4	5
**B13.**	Hopelessness about the future	1	2	3	4	5
**B14.**	Inability to feel joy	1	2	3	4	5
**B15.**	Feeling easily irritated or annoyed	1	2	3	4	5
**B16.**	Feeling anxious or fearful	1	2	3	4	5
**B17.**	Feeling tense or unable to relax	1	2	3	4	5
**B18.**	Excessive worry about several things	1	2	3	4	5
**B19.**	Feeling so anxious or restless that it is hard to sit still	1	2	3	4	5
**B20.**	Being easily startled	1	2	3	4	5
**B21.**	Sudden panic attacks with palpitations, shortness of breath, faintness or other distressing bodily sensations	1	2	3	4	5
**B22.**	Fear of being away from home alone	1	2	3	4	5
**B23.**	Feeling afraid in public spaces or on the street	1	2	3	4	5
**B24.**	Fear of fainting in public	1	2	3	4	5
**B25.**	Fear of travelling by bus, tram, train or car	1	2	3	4	5
**B26.**	Fear of being the centre of attention	1	2	3	4	5
**B27.**	Fear of interacting with strangers	1	2	3	4	5
**B28.**	Fatigue or loss of energy	1	2	3	4	5
**B29.**	Diminished attention span or ability to concentrate	1	2	3	4	5
**B30.**	Resting does not restore strength	1	2	3	4	5

		Not at all	Rarely	Some-times	Often	Constantly
**B31.**	Being easily fatigued	1	2	3	4	5
**B32.**	Difficulty falling asleep	1	2	3	4	5
**B33.**	Restless or disturbed sleep	1	2	3	4	5
**B34.**	Waking up too early	1	2	3	4	5
**B35.**	Deliberate self-harm (such as intentionally cutting your skin or causing pain, hitting yourself)	1	2	3	4	5

How much (or how often) have the following problems or complaints bothered you <u>during the last four (4) weeks?</u>

		Not at all	Rarely	Some-times	Often	Constantly
**B36.**	Sleeping less than usual, but still have a lot of energy	1	2	3	4	5
**B37.**	Starting lots more projects than usual or doing more risky things than usual	1	2	3	4	5
**B38.**	Unexplained aches and pains (e.g., head, back, joints, abdomen, legs)	1	2	3	4	5
**B39.**	Feeling that your illnesses are not being taken seriously enough	1	2	3	4	5
**B40.**	Hearing things other people couldn’t hear, such as voices even when no one was around	1	2	3	4	5
**B41.**	Feeling that someone could hear your thoughts, or that you could hear what another person was thinking	1	2	3	4	5
**B42.**	Problems with memory (e.g., learning new information) or with location (e.g., finding your way home)	1	2	3	4	5
**B43.**	Unpleasant thoughts, urges, or images that repeatedly enter your mind	1	2	3	4	5
**B44.**	Feeling driven to perform certain behaviors or mental acts over and over again	1	2	3	4	5
**B45.**	Feeling detached or distant from yourself, your body, your physical surroundings, or your memories	1	2	3	4	5

How much do the following statements apply to you? Please select the most applicable answer.

		Completely false	Mostly false	Neither true nor false	Mostly true	Completely true
**B46.**	Most of the time I feel lively and energetic.	1	2	3	4	5
**B47.**	Most of the time I feel attentive and alert.	1	2	3	4	5
**B48.**	I am hopeful and enthusiastic about the future.	1	2	3	4	5

B49. Sometimes things happen to people that are particularly frightening or traumatic. Such events can include natural disasters and other catastrophes, wars, serious accidents and fires, a serious illness, being placed under intensive care, sexual or physical assault or abuse, witnessing a murder, suicide or injuries and the sudden death of someone close. **Have you experienced such events over the past three (3) months?**

1 No → *Proceed to question B50*   2 Yes

Below is a list of problems and complaints that people sometimes have in response to stressful experiences. Please indicate how much each problem has bothered you during the last four (4) weeks.

		Not at all	Rarely	Sometimes	Often	Constantly
**B49a.**	Repeated, disturbing memories, thoughts or images of a stressful experience	1	2	3	4	5
**B49b.**	Feeling very upset when something reminded you of a stressful experience	1	2	3	4	5
**B49c.**	Avoiding activities or situations because they reminded you of a stressful experience	1	2	3	4	5
**B49d.**	Being watchful or easily startled	1	2	3	4	5

Next, we want to know the importance of food and eating in your life over the past three (3) months.

B50. Have you spent a considerable amount of time thinking about food and your weight?

1 No   2 Yes

B51. Have you considerably limited your diet over the past three months?

1 No 2 Yes

B52. Have you been binge eating (eating more than usual) over the past three months?

1 No → *Proceed to question B53* 2 Yes

B52a. During these binges, have you felt that you cannot control your eating?

1 No 2 Yes

B53. Have you deliberately made yourself vomit, used laxatives or appetite suppressants to control your weight over the past three months?

1 No 2 Yes

In the following section we will be asking about your general health and health behaviour.

C1. How would you assess your current state of health?

1 Very good
2 Good
3 Average
4 Poor
5 Very poor

C2. How concerned have you been about your health over the past three (3) months?

1 Not at all
2 A little
3 To some extent
4 A lot
5 Very much

C3. Over the past three (3) months, how often in your leisure time have you been active (playing sports, doing gardening, high-speed cycling or brisk walking, etc.) for at least 30 min at a time so that you are slightly out of breath or sweating?

1 Never
2 Once a month or less
3 2–3 times per month
4 Once a week
5 2–3 times a week
6 4–6 times a week
7 Every day

C4. How many minutes do you walk or ride a bicycle on a regular day?

1 Less than 15 minutes a day
2 15–30 minutes a day
3 30–60 minutes a day
4 More than 60 minutes a day

C5. How much physical effort does your everyday work (incl. studies at school or at university) require?

1 very little (mainly sitting)

2 some (mainly moving around)

3 average (lifting, carrying light loads)

4 a lot (carrying heavy loads, climbing)

5 I don't work/study

C6. Have you smoked <u>in the past three (3) months</u>?

1 No → **C6a. Have you ever smoked?**

1 I have never smoked

2 I quit more than 6 months ago

3 I quit less than 6 months ago

2 Yes → **C6b. What characterises your smoking?** Select all applicable responses.

1 I smoke cigarettes/cigars/a pipe daily

2 I smoke e-cigarettes or other smoke-free products daily

3 I smoke cigarettes/cigars/a pipe occasionally

4 I smoke e-cigarettes or other smoke-free products occasionally

C7. How often have you had a drink containing alcohol <u>over the past three (3) months</u>?

1 Never → *Proceed to question C17*

2 Monthly or less

3 2–4 times per month

4 2–3 times per week

5 4 or more times per week

C8. How many units of alcohol did you usually consume at one time <u>in the past three (3) months</u>?

1 1–2

2 3–4

3 5–6

4 7–9

5 10+

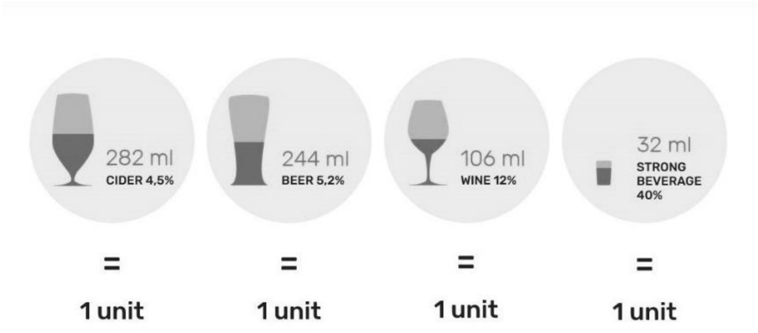

		Never	Less than once a month	Once a month	Once a week	Every day or almost every day
**C9.**	How often have you had 6 or more units on a single occasion?	1	2	3	4	5
**C10.**	How often over the past three (3) months have you found that you were not able to stop drinking once you had started?	1	2	3	4	5
**C11.**	How often over the past three (3) months have you failed to do what was normally expected of you because of drinking?	1	2	3	4	5
**C12.**	How often over the past three (3) months have you needed a first drink in the morning to get yourself going after a heavy drinking session?	1	2	3	4	5
**C13.**	How often over the past three (3) months have you had a feeling of guilt and remorse after drinking?	1	2	3	4	5
**C14.**	How often over the past three (3) months have you been unable to remember what happened the night before because of your drinking?	1	2	3	4	5

C15. Have you or someone else been injured because of your drinking?

1 No

2 Yes, but not during the last three (3) months

3 Yes, during the last three (3) months

C16. Has a relative, friend, doctor, or other health care worker been concerned about your drinking or suggested you cut down?

1 No

2 Yes, but not during the last three (3) months

3 Yes, during the last three (3) months

C17. How often have you used narcotic substances over the past three (3) months? Narcotic substances include cannabis, cocaine or crack, 'party drugs' (such as ecstasy), hallucinogens (such as LSD), heroine, solvents or inhalants (such as glue) or methamphetamine (such as speed).

1 Never

2 Once a month or less

3 2–4 times a month

4 2–3 times a week

5 4 or more times a week

C18. How many hours a day have you slept <u>in the past three (3) months</u>? Include naps in your calculation.

…… hours and …… minutes on a weekday

…… hours and …… minutes at the weekend

How many portions of food from the following food groups have you usually eaten a day <u>over the past four (4) weeks</u>?

C19. Fruit/berries (incl. boiled/cooked/canned and fresh, excluding juice):
*1 portion is about 1 handful (~100 grams).

...... portions a day

C20. Vegetables (incl. boiled/cooked/canned and fresh, excluding potatoes):
*1 portion is about 1 handful (~100 grams).

...... portions a day

C21. Confectionery (e.g. biscuits, sweets, chocolate, halwa, ice-cream, pastry, etc.):
*1 portion is about 40 kcal or ~10 g sweets.

...... portions a day

(1 portion = 1 sweet or 2 squares of chocolate or 1 biscuit or 1/4 of curd snack or 1/5 of ice-cream/a piece of pastry or 1/10 of a piece of cake)

C22. Savoury snacks (e.g. potato chips or corn tortillas, popcorn, salted peanuts):
*1 portion is about 40 kcal or ~10 g of savoury snacks

...... portions a day

(1 portion = 1dl or a handful of crisps/garlic bread/popcorn or 1tbsp of salted peanuts)

C23. Sweet soft drinks and fruit drinks (incl. flavoured water and non-alcoholic drinks with sugar or sweeteners):
*1 portion is ~ 100 grams of sweet drinks.

...... portions a day

Next, we will ask you to respond to questions about your relationships with your family and those close to you.

C24. Has the pandemic had an impact on your relationships with your family members?

1 No, it has not had an impact

2 Yes, it has had an impact, the relationships have become more stressful

3 Yes, it has had an impact, the relationships have become closer

C25. How often does emotional abuse (swearing or yelling at you, insulting, etc.) occur in your family?

1 Never
2 Very rarely
3 It has occurred occasionally
4 Quite often
5 Almost every day

C26. Have you experienced domestic abuse of an emotional, sexual or physical nature <u>over the past three (3) months</u>? Domestic abuse is any mental, physical or sexual abuse which occurs between people who are or have at some point been in an intimate relationship or legally bound, or who are related by blood.

1 No
2 Yes
3 Don't wish to say

The following questions are about using smart devices and social media in your everyday life.

C27. How many hours on a regular day do you use electronic devices (computer, tablet, smartphone, etc.) for work?

1 Not at all
2 Less than one hour a day
3 1–2 hours a day
4 2–4 hours a day
5 4–6 hours a day
6 More than 6 hours a day

C28. How many hours on a regular day do you use electronic devices (TV, computer, tablet, smartphone, etc.) for leisure activities (incl. YouTube and games)?

1 Not at all
2 Less than one hour a day
3 1–2 hours a day
4 2–4 hours a day
5 4–6 hours a day
6 More than 6 hours a day

C29. How many hours on a regular day do you use electronic devices (computer, tablet, smartphone, etc.) for non-work communication?

1 Not at all
2 Less than one hour a day
3 1–2 hours a day
4 2–4 hours a day
5 4–6 hours a day
6 More than 6 hours a day

The following questions are about your social media use.

C30. Do you use social media? (e.g. Facebook, Twitter, Instagram)

1 No → *proceed to question C35*
2 Yes

		Never	Rarely	Some-times	Often	Very often
**C31.**	How often do you find that you spend more time with social media than you intended?	1	2	3	4	5
**C32.**	How often have you neglected your obligations or chores because of using social media?	1	2	3	4	5
**C33.**	How often do you feel preoccupied with using social media?	1	2	3	4	5
**C34.**	How often have people told you that you use social media too much?	1	2	3	4	5

Please read the following statements and indicate how much you agree or disagree with each statement.

		Agree strongly	Agree some-what	Disagree some-what	Disagree strongly
**C35.**	When I am in great mood, I tend to get into situations that could cause me problems.	1	2	3	4
**C36.**	I tend to lose control when I am in a great mood.	1	2	3	4
**C37.**	Others are shocked or worried about the things I do when I am feeling very excited.	1	2	3	4
**C38.**	I tend to act without thinking when I am really excited.	1	2	3	4
**C39.**	When I feel bad, I will often do things I later regret in order to make myself feel better now.	1	2	3	4
**C40.**	Sometimes when I feel bad, I can’t seem to stop what I am doing even though it is making me feel worse.	1	2	3	4
**C41.**	When I am upset I often act without thinking.	1	2	3	4
**C42.**	When I feel rejected, I will often say things that I later regret.	1	2	3	4
**C43.**	I have a hard time making it through stressful events.	1	2	3	4
**C44.**	I tend to bounce back quickly after setbacks in my life.	1	2	3	4

C45. How do you see yourself? Are you generally a person who is fully prepared to take risks or do you try to avoid taking risks? Please rate on a scale of 0 to 10, where the value 0 means 'not at all willing to take risks' and the value 10 means 'very willing to take risks'.

0 1 2 3 4 5 6 7 8 9 10

The following questions are about your work or studies and the stress these cause. *If you don't work or study, please proceed to question C49.*

C46. How many hours a week do you work/study?

…… hours

C47. Do you work remotely? / Are you enrolled in distance learning?

1 No, I don't have such a possibility

2 No, but I would have the possibility

3 Yes

C48. How often have you felt completely exhausted because of work/studies?

1 Never

2 Rarely

3 Sometimes

4 Often

5 Very often

C49. How often have you felt completely exhausted because of household responsibilities?

1 Never

2 Rarely

3 Sometimes

4 Often

5 Very often

The following questions are about your everyday life and how you have been dealing with it <u>since January 2021</u>.

D1. Do you face a heightened risk of contact with COVID-19 infected people due to your profession?

1 No

2 Yes, I have worked as a doctor, nurse or pharmacist

3 Yes, I have worked as a caregiver

4 Yes, I have worked as a police officer

5 Yes, I have been a service worker

6 Yes, I have worked as a public transport driver

7 Yes, I have worked as a teacher

8 Yes, other. Please specify: ………………………………..……………………………

D2. Have you been diagnosed with coronavirus?

1 No, I have not been diagnosed with coronavirus

2 Yes, I have been diagnosed with coronavirus

D3. Do you feel you have been subject to unfavourable, negative or prejudiced attitude due to your possible contact with coronavirus?

1 No 2 Yes

D4. Have you been vaccinated against COVID-19? Respond "yes" also when you have only been vaccinated once.

1 No 2 Yes → *Proceed to question D6*

D5. Are you planning to get vaccinated against COVID-19 if you have the opportunity?

1 Yes, definitely

2 I haven't decided but most likely yes

3 I don't know

4 I haven't decided but probably not

5 Definitely not

D6. Has your employment status changed since January 2021?

1 It has not changed

2 Yes, I am working more than before

3 Yes, I am working less than before

4 Yes, I decided to remain at home with the children

5 Yes, I decided to retire

6 Yes, I was laid off or became unemployed

7 Other. Please specify: ……………………………………………………….………………

D7. How many times <u>since the beginning of the COVID epidemic</u> have you had to self-isolate due to the virus (incl. testing positive, being a close contact or arriving from abroad)? Write the number of self-isolation cases <u>since spring 2020</u>. Count consecutive self-isolations as separate cases. If you haven't had to self-isolate, write 0 *and proceed to question D9.*

…... times

D8. Which statements characterise your behaviour during the <u>last</u> period of self-isolation? Mark all applicable statements.

1 I stayed at home during the whole self-isolation period

2 I left house only to spend time outdoors and visit essential services (shop, pharmacy or medical centre)

3 I left house to go to work or school

4 I left house to meet friends or go to gym or attend hobby clubs

5 I left house to visit leisure attractions (cafés, restaurants, cinemas, theatres, concerts, etc.)

6 I didn’t change anything in my life during the self-isolation period

Please rate how much the following measures to prevent the spread of coronavirus currently in use and the characteristics of the situation have caused you stress.

		Not applicable	Caused no stress	Caused some stress	Caused significant stress
**D9.**	Restrictions on shopping centres	1	2	3	4
**D10.**	Distance learning in primary school	1	2	3	4
**D11.**	Distance learning in basic and secondary schools and universities	1	2	3	4
**D12.**	Limited access to childcare / kindergartens	1	2	3	4
**D13.**	Reorganisation of work, e.g. remote working	1	2	3	4
**D14.**	Restrictions on entertainment establishments (such as theatres, cinemas, museums, exhibitions, concerts, cafés and restaurants)	1	2	3	4
**D15.**	Restrictions on organising events, including official and family events (such as graduations, birthday parties, weddings and funerals)	1	2	3	4
**D16.**	Restrictions on sports facilities (such as fitness clubs, stadiums, outdoor gyms and playgrounds)	1	2	3	4
**D17.**	Restrictions on visiting churches or other religious establishments	1	2	3	4
**D18.**	The 2+2 rule in public spaces	1	2	3	4
**D19.**	The compulsory isolation of those infected and their contacts	1	2	3	4
**D20.**	The closing of borders and introduction of travel restrictions	1	2	3	4
**D21.**	Self-isolation when travelling from abroad	1	2	3	4
**D22.**	Restrictions on alcohol sales in bars/restaurants	1	2	3	4
**D23.**	Temporary interruptions to planned medical treatment	1	2	3	4
**D24.**	Reduced access to social services (family care, personal care or home care services)	1	2	3	4
**D25.**	Visiting restrictions (including friends, elderly family members and loved ones in hospitals and nursing homes)	1	2	3	4
**D26.**	Economic or labour market uncertainty	1	2	3	4

		Not applicable	Caused no stress	Caused some stress	Caused significant stress
**D27.**	Limited opportunities for social interaction	1	2	3	4
**D28.**	Recommended / required mask wearing in public spaces	1	2	3	4
**D29.**	Ambiguity around restrictions and recommendations	1	2	3	4
**D30.**	Short notice for announcing or removing restrictions	1	2	3	4
**D31.**	Ambiguities around the vaccination schedule	1	2	3	4
**D32.**	Lack of clarity around when the pre-COVID situation would return	1	2	3	4
**D33.**	Risk of falling seriously ill due to the corona virus	1	2	3	4
**D34.**	Risk of a person close to me falling seriously ill due to the corona virus	1	2	3	4
**D35.**	Risk that I will cause someone fall seriously ill due to the corona virus	1	2	3	4

D36. With all things considered, how stressed do you currently feel due to the coronavirus crisis?

1 Not at all
2 A little
3 Somewhat
4 A lot
5 Very much

D37. What have you done <u>in the past four (4) weeks</u> to prevent yourself or others from becoming infected with coronavirus? Select all of the measures you have taken.

1 Regularly washing and disinfecting your hands
2 Covering your mouth and nose when coughing or sneezing
3 Wearing a mask or a visor
4 Keeping a safe distance from others
5 Avoiding events and gatherings
6 Avoiding shopping centres and grocery stores
7 Staying home at any sign of illness
8 Getting tested for coronavirus
9 Getting vaccinated against coronavirus
10 Avoiding public transport
11 Avoiding indoor public spaces

12 Staying at home

13 None of the above

14 Other. Please specify: ……………………………………………………….………………

D38. Please name three (3) most important activities that have helped you improve your mood or sense of well-being during the corona virus crisis.

1 ……………………………………………………….………………………….

2 ………………………………………………………………………….…….

3 ……………………………………………………………………….………..…

Today's date [c] |__|__| |__|__| 2021
Day Month

You have now reached the end of the questionnaire. Please make sure that you have answered all the questions.

Thank you very much for taking the time to complete the questionnaire!

Do you wish to be entered in the gift voucher prize draw? 1 No 2 Yes

If you have any additional information that you would like to share with us, please do so in the space below.

---

[a] In web survey, only displayed to wave 1 non-respondents

[b] Only in web survey

[c] Only in postal survey
