# Supplementary material for: Estonian National Mental Health Study: Design and methods for a registry‐linked longitudinal survey
Source: Brain Behav. 2023 Jun 5;13(8):e3106. doi: 10.1002/brb3.3106 (PMC10454261; doi:10.1002/brb3.3106)
Supplement: Supplementary file 3 — Additional file 3. Wave 3 questionnaire for adults (PDF) [file BRB3-13-e3106-s001.pdf]

ESTONIAN NATIONAL MENTAL HEALTH STUDY: WAVE 3 QUESTIONNAIRE FOR ADULTS

Thank you for agreeing to participate in the third wave of the Estonian National Mental Health Study. The following questionnaire is about various aspects of your well-being. **To choose an answer option, circle the number next to the appropriate option or write it in the designated space. Every response is very important to us.** We assure that all your answers are treated with full confidentiality.

First, we will ask you some questions about your general life situation.

A1. Sex 1 Male 2 Female

A2. Date of birth |__|__| |__|__| |__|__|__|__|
Day Month Year

A3. What is your current marital status?

1 Single (never been married/cohabited)

2 Married or cohabiting

3 In a steady relationship but do not live together

4 Divorced/separated

5 Widowed

A4. What is the highest level of education you have completed?

1 Primary education (up to 6 years)

2 Basic education

3 Basic education with vocational training

4 Secondary education

5 Vocational secondary education

6 Higher vocational education

7 Undergraduate degree (Bachelor's)

8 Postgraduate degree (Master's, PhD/candidate)

A5. Which of the following is the most accurate description of your current employment status? Select one primary status.

1 I am studying or doing an unpaid internship → *Proceed to question A7*

2 I am employed/working as a contractor → *Proceed to question A7*

3 I am an entrepreneur → *Proceed to question A7*

4 I am registered as unemployed

5 I am unemployed and not actively seeking employment

6 I am an old-age pensioner → *Proceed to question A7*

7 I have been declared incapacitated for work → *Proceed to question A7*

8 I am on parental leave → *Proceed to question A7*

9 I am a home-maker → *Proceed to question A7*

10 I am in military service → *Proceed to question A7*

11 I am the caregiver to a close relative → *Proceed to question A7*

12 Other. Please specify: ………………………………………………….…………………

A6. How many months ago did you last work?

1 ...... months ago (Write the number of months if less than a year.)

2 More than 12 months ago

A7. What else do you do in addition to your primary activity? Select all applicable responses.

1 I do nothing else

2 I am studying or doing an unpaid internship

3 I am employed/working as a contractor

4 I am an entrepreneur

5 I am an old-age pensioner

6 I have been declared incapacitated for work

7 I am the caregiver to a close relative

8 Other. Please specify: …………………………..………………………………………...

A8. Has your employment status changed <u>over the past six (6) months</u>?

1 It has not changed

2 Yes, I am working more than before

3 Yes, I am working less than before

4 Yes, I decided to remain at home with the children

5 Yes, I decided to retire

6 Yes, I was laid off or became unemployed

7 Other. Please specify: ……………………………………………………..………………

A9. What is your current or most recent occupation? Select one primary occupation.

1 I have never worked

2 Legislator, senior official, manager (public official, director, executive, etc.)

3 Professional (engineer, physician, software developer, lecturer, teacher, researcher, creative professional, etc.)

4 Associate professional (technician, inspector, nurse, real estate agent, social worker, etc.)

5 Official, customer service representative (secretary, clerk, administrator, etc.)

6 Service or sales staff (guide, chef, cashier, babysitter, police officer, prison official, salesperson, janitor, etc.)

7 Skilled labourer in agriculture, fishery, forestry or hunting (gardener, breeder, fisher, farmer, etc.)

8 Skilled worker (miner, carpenter, electrician, printing worker, tailor, craftsman, etc.)

9 Device or machine operator (operator, assembler, bus driver, crane operator, etc.)

10 Unskilled worker (guard, cleaner, street vendor, agricultural worker, transport worker, etc.)

11 Professional soldier

A10. What is your average monthly net income for the last 12 months? (net income from all sources, including family allowance, pensions and rental income)

1 No income
2 Up to 450 euros
3 451–650 euros
4 651–850 euros
5 851–1100 euros
6 1101–1400 euros
7 1401–1700 euros
8 1701–2000 euros
9 2001–2500 euros
10 2501 euros or more

A11. What is the current financial situation of your household? A household is generally comprised of people living in the same dwelling who share food or a budget.

1 We have money to spare
2 We have enough money to get by
3 We are barely making ends meet
4 We do not have enough money to cover unavoidable costs (such as utility bills)
5 Cannot say

A12. How many people live in your household?

1 I live alone → *Proceed to question A15*
2 There are ...... people in addition to me

A13. How many children live in your household? Include yourself, if applicable. Mark 0 if no children of the given age live in your household.

.... children under 7 years of age
…... children aged 7–17
…... children aged 18–19 studying in a general education school

A14. Who currently belong to your household? Mark everyone you live with.

1 Spouse/partner
2 One parent
3 Both parents
4 One or more in-laws
5 One or more grandparents
6 One or more siblings
7 One or more children (including adult children)
8 One or more grandchildren
9 Other. Please specify: ……………………..…………

A15. Does anyone in your household require constant care due to an illness or a medical condition? Select all applicable responses.

1 No one requires care

2 I require care myself

3 Someone else in the household requires care

A16. Where is your current residence?

1 Harju County, excluding Tallinn

2 Tallinn

3 Hiiu County

4 Ida-Viru County

5 Jõgeva County

6 Järva County

7 Lääne County

8 Lääne-Viru County

9 Põlva County

10 Pärnu County

11 Rapla County

12 Saare County

13 Tartu County, excluding Tartu

14 Tartu

15 Valga County

16 Viljandi County

17 Võru County

18 I do not reside in Estonia. Please specify your country of residence:

....

A17. What type of settlement do you currently reside in?

1 A settlement with a population of less than 1000 or in the countryside

2 A settlement with a population of 1000–10,000

3 A settlement with a population of more than 10,000

The following questions are about your well-being, how you feel and your mental health.

B1. How much problem have feeling sad, low, or depressed caused you during <u>the last four (4) weeks</u>?

1 None

2 Mild

3 Moderate

4 Severe

5 Extreme

Please rate your satisfaction with the following aspects of your life:

		Very satisfied	Quite satisfied	Not particularly satisfied	Not satisfied at all
**B2.**	Life in general	1	2	3	4
**B3.**	Your financial situation	1	2	3	4

		Very satisfied	Quite satisfied	Not particularly satisfied	Not satisfied at all	
**B4.**	Family relations	1	2	3	4	
**B5.**	Friendships	1	2	3	4	
**B6.**	Work	1	2	3	4	5 I do not work

B7. Have you been diagnosed with a mental disorder (such as depression, anxiety or alcoholism) <u>over the past twelve (12) months</u>?

1 No

2 Yes

3 Don't know

4 Prefer not to answer

Carefully read the following list of problems and complaints that people sometimes experience. Please indicate how much each one has bothered you <u>during the last four (4) weeks</u>.

		Not at all	Rarely	Some-times	Often	Constantly
**B8.**	Sadness	1	2	3	4	5
**B9.**	Lack of interest in things	1	2	3	4	5
**B10.**	Feeling of worthlessness	1	2	3	4	5
**B11.**	Self-accusations	1	2	3	4	5
**B12.**	Recurrent thoughts of death or suicide	1	2	3	4	5
**B13.**	Feeling lonely	1	2	3	4	5
**B14.**	Hopelessness about the future	1	2	3	4	5
**B15.**	Inability to feel joy	1	2	3	4	5
**B16.**	Feeling easily irritated or annoyed	1	2	3	4	5
**B17.**	Feeling anxious or fearful	1	2	3	4	5
**B18.**	Feeling tense or unable to relax	1	2	3	4	5
**B19.**	Excessive worry about several things	1	2	3	4	5
**B20.**	Feeling so anxious or restless that it is hard to sit still	1	2	3	4	5
**B21.**	Being easily startled	1	2	3	4	5

		Not at all	Rarely	Some-times	Often	Constantly
**B22.**	Sudden panic attacks with palpitations, shortness of breath, faintness or other distressing bodily sensations	1	2	3	4	5
**B23.**	Fear of being away from home alone	1	2	3	4	5
**B24.**	Feeling afraid in public spaces or on the street	1	2	3	4	5
**B25.**	Fear of fainting in public	1	2	3	4	5
**B26.**	Fear of travelling by bus, tram, train or car	1	2	3	4	5
**B27.**	Fear of being the centre of attention	1	2	3	4	5
**B28.**	Fear of interacting with strangers	1	2	3	4	5
**B29.**	Fatigue or loss of energy	1	2	3	4	5
**B30.**	Diminished attention span or ability to concentrate	1	2	3	4	5
**B31.**	Resting does not restore strength	1	2	3	4	5
**B32.**	Being easily fatigued	1	2	3	4	5
**B33.**	Difficulty falling asleep	1	2	3	4	5
**B34.**	Restless or disturbed sleep	1	2	3	4	5
**B35.**	Waking up too early	1	2	3	4	5
**B36.**	Deliberate self-harm (such as intentionally cutting your skin or causing pain, hitting yourself)	1	2	3	4	5

How much (or how often) have the following problems or complaints bothered you <u>during the last four (4) weeks?</u>

		Not at all	Rarely	Some-times	Often	Constantly
**B37.**	Sleeping less than usual, but still have a lot of energy	1	2	3	4	5
**B38.**	Starting lots more projects than usual or doing more risky things than usual	1	2	3	4	5

		Not at all	Rarely	Some-times	Often	Constantly
**B39.**	Unexplained aches and pains (e.g., head, back, joints, abdomen, legs)	1	2	3	4	5
**B40.**	Feeling that your illnesses are not being taken seriously enough	1	2	3	4	5
**B41.**	Hearing things other people couldn't hear, such as voices even when no one was around	1	2	3	4	5
**B42.**	Feeling that someone could hear your thoughts, or that you could hear what another person was thinking	1	2	3	4	5
**B43.**	Problems with memory (e.g., learning new information) or with location (e.g., finding your way home)	1	2	3	4	5
**B44.**	Unpleasant thoughts, urges, or images that repeatedly enter your mind	1	2	3	4	5
**B45.**	Feeling driven to perform certain behaviors or mental acts over and over again	1	2	3	4	5
**B46.**	Feeling detached or distant from yourself, your body, your physical surroundings, or your memories	1	2	3	4	5

How much do the following statements apply to you? Please select the most applicable answer.

		Completely false	Mostly false	Neither true nor false	Mostly true	Completely true
**B47.**	Most of the time I feel lively and energetic.	1	2	3	4	5
**B48.**	Most of the time I feel attentive and alert.	1	2	3	4	5
**B49.**	I am hopeful and enthusiastic about the future.	1	2	3	4	5

Next, we want to know the importance of food and eating in your life <u>over the past three (3) months</u>.

B50. Have you spent a considerable amount of time thinking about food and your weight?

1 No 2 Yes

B51. Have you considerably limited your diet <u>over the past three months</u>?

1 No 2 Yes

B52. Have you been binge eating (eating more than usual) <u>over the past three months</u>?

1 No → *Proceed to question B54* 2 Yes

B53. During these binges, have you felt that you cannot control your eating?

1 No 2 Yes

B54. Have you deliberately made yourself vomit, used laxatives or appetite suppressants to control your weight <u>over the past three months</u>?

1 No 2 Yes

Carefully read the following statements and indicate to what extent you agree with each statement.

		**Completely agree**	**Rather agree**	**Rather disagree**	**Completely disagree**
**B55.**	I am fascinated by dates.	1	2	3	4
**B56.**	I usually notice car number plates or similar strings of information.	1	2	3	4
**B57.**	I find it easy to "read between the lines" when someone is talking to me.	1	2	3	4
**B58.**	I find social situations easy.	1	2	3	4
**B59.**	If I try to imagine something, I find it very easy to create a picture in my mind.	1	2	3	4
**B60.**	It does not upset me if my daily routine is disturbed.	1	2	3	4
**B61.**	When I'm reading a story, I can easily imagine what the characters might look like.	1	2	3	4
**B62.**	I find it difficult to work out people's intentions.	1	2	3	4
**B63.**	I find it hard to make new friends.	1	2	3	4
**B64.**	I enjoy doing things spontaneously.	1	2	3	4

The following questions are about your childhood environment and your exposure to possible traumatic events until the age of 18 years. Such experiences are frequent and can sometimes have a long-term impact on a person's health and well-being. We will present a series of statements about difficult life events and we ask you to indicate all that apply to you.

		Yes	No	Cannot say	Prefer not to answer
**B65.**	Did you feel that you didn't have enough to eat, had to wear dirty clothes, or had no one to protect or take care of you?	1	2	3	4
**B66.**	Did you lose a parent through divorce, abandonment, death, or other reason?	1	2	3	4
**B67.**	Did you live with anyone who was depressed, mentally ill, or attempted suicide?	1	2	3	4
**B68.**	Did you live with anyone who had a problem with drinking or using drugs?	1	2	3	4
**B69.**	Did your parents or adults in your home ever hit, punch, beat, or threaten to harm each other?	1	2	3	4
**B70.**	Did you live with anyone who went to jail or prison?	1	2	3	4
**B71.**	Did a parent or adult in your home ever swear at you, insult you, or put you down?	1	2	3	4
**B72.**	Did a parent or adult in your home ever hit, beat, kick, or physically hurt you in any way?	1	2	3	4
**B73.**	Did you feel that no one in your family loved you or thought you were special?	1	2	3	4
**B74.**	Did you experience unwanted sexual contact?	1	2	3	4

Next, we will ask about events that took place in adult years (18 years of age and older) because these events can equally have long-term impact on someone's health and well-being.

		Never	Yes, but not within the last 12 months	Yes, within the last 12 months	Yes, within the last month	Prefer not to answer
**B75.**	Have you experienced serious financial difficulties over a longer period of time when you didn't have money to pay utility bills and buy food?	1	2	3	4	5

		Never	Yes, but not within the last 12 months	Yes, within the last 12 months	Yes, within the last month	Prefer not to answer
**B76.**	Have you ever experienced domestic abuse of an emotional, sexual or physical nature? **Domestic abuse is any mental, physical or sexual abuse which occurs between people who are or have at some point been in an intimate relationship or legally bound, or who are related by blood.*	1	2	3	4	5
**B77.**	Have you ever been sexually assaulted either by someone you knew or didn't know, who is not part of your family?	1	2	3	4	5
**B78.**	Have you ever been subject to a violent crime (physical assault, robbery, etc.)? Please exclude domestic abuse.	1	2	3	4	5
**B79.**	Have you ever experienced a serious accident that was or which you perceived as life-threatening (car accident, fire, etc.)?	1	2	3	4	5
**B80.**	Have you ever witnessed a sudden violent death (e.g., murder, suicide, aftermath of an accident)?	1	2	3	4	5
**B81.**	Have you ever been diagnosed with a life-threatening illness?	1	2	3	4	5
**B82.**	Has anyone close to you ever died unexpectedly?	1	2	3	4	5
**B83.**	Have you ever experienced a life threatening or extremely stressful event (e.g. natural disaster, being in a war zone, etc.)?	1	2	3	4	5

If you answered "no" to questions B65–B74 and "never" to questions B75–B83, proceed to question C1.

Below is a list of problems and complaints that people sometimes have in response to stressful experiences. Please indicate how much each problem has bothered you <u>during the last four (4) weeks.</u>

		Not at all	Rarely	Some-times	Often	Constantly
**B84.**	Repeated, disturbing memories, thoughts or images of a stressful experience	1	2	3	4	5
**B85.**	Feeling very upset when something reminded you of a stressful experience	1	2	3	4	5
**B86.**	Avoiding activities or situations because they reminded you of a stressful experience	1	2	3	4	5
**B87.**	Being watchful or easily startled	1	2	3	4	5

In the following section we will be asking about your general health and health behaviour.

C1. How tall are you? (without shoes)

……… cm

C2. How much do you weigh? (without clothes) If you are pregnant, note your weight prior to your pregnancy.

……… kg

C3. How would you assess your current state of health?

1 Very good
2 Good
3 Average
4 Poor
5 Very poor

C4. Do you have any long-standing (chronic) illness or health problem?

1 No
2 Yes

C5. How concerned have you been about your health <u>over the past six (6) months</u>?

1 Not at all
2 A little
3 To some extent
4 A lot
5 Very much

Under each heading, please select the answer option that best describes your health <u>today</u>.

C6. Mobility:

1 I have no problems in walking about
2 I have some problems in walking about
3 I am confined to bed

C7. Self-care:

1 I have no problems with self-care
2 I have some problems washing or dressing myself
3 I am unable to wash or dress myself

C8. Usual activities (e.g. work, study, housework, family or leisure activities):

1 I have no problems with performing my usual activities
2 I have some problems with performing my usual activities
3 I am unable to perform my usual activities

C9. Pain / discomfort:

1 I have no pain or discomfort
2 I have moderate pain or discomfort
3 I have severe pain or discomfort

C10. Anxiety / depression:

1 I am not anxious or depressed
2 I am moderately anxious or depressed
3 I am extremely anxious or depressed

C11. <u>Over the past three (3) months</u>, how often in your leisure time have you been active (playing sports, doing gardening, high-speed cycling or brisk walking, etc.) for at least 30 min at a time so that you are slightly out of breath or sweating?

1 Never
2 Once a month or less
3 2–3 times per month
4 Once a week
5 2–3 times a week
6 4–6 times a week
7 Every day

C12. Have you smoked <u>in the past three (3) months</u>?

1 No → **C13a. Have you ever smoked?**

1 I have never smoked
2 I quit more than 6 months ago
3 I quit less than 6 months ago

2 Yes → **C13b. What characterises your smoking?** Select all applicable responses.

1 I smoke cigarettes/cigars/a pipe daily
2 I smoke e-cigarettes or other smoke-free products daily
3 I smoke cigarettes/cigars/a pipe occasionally
4 I smoke e-cigarettes or other smoke-free products occasionally

C14. How often have you had a drink containing alcohol <u>over the past three (3) months</u>?

1 Never → *Proceed to question C17*
2 Monthly or less
3 2–4 times per month
4 2–3 times per week
5 4 or more times per week

C15. How many units of alcohol did you usually consume at one time <u>in the past three (3) months</u>?

1 1–2
2 3–4
3 5–6
4 7–9
5 10+

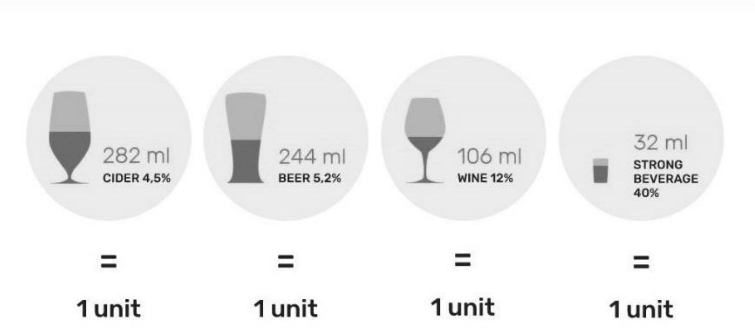

C16. How often have you had 6 or more units on a single occasion?

1 Never
2 Less than once a month
3 Once a month
4 Once a week
5 Every day or almost every day

C17. How often have you used narcotic substances <u>over the past three (3) months</u>? Narcotic substances include cannabis, cocaine or crack, 'party drugs' (such as ecstasy), hallucinogens (such as LSD), heroine, solvents or inhalants (such as glue) or methamphetamine (such as speed).

1 Never
2 Once a month or less
3 2–4 times a month
4 2–3 times a week
5 4 or more times a week

C18. On average, how many hours a day have you slept <u>in the past three (3) months</u>? Include naps in your calculation.

…… hours and …… minutes on a weekday

…… hours and …… minutes at the weekend

C19. How often have you recently felt that difficulties were piling up so high that you could not overcome them?

1 Very rarely
2 Quite rarely
3 Sometimes
4 Quite often
5 Very often

C20. How often does emotional abuse (swearing or yelling at you, insulting, etc.) occur in your family?

1 Never
2 Very rarely
3 It has occurred occasionally
4 Quite often
5 Almost every day

Next questions are about your personality traits. Please rate how much the following statements apply to you.

		Complete-ly false	Mostly false	Some-what false	Some-what true	Mostly true	Complete-ly true
**C21.**	I know how to comfort others.	1	2	3	4	5	6
**C22.**	I keep things tidy.	1	2	3	4	5	6
**C23.**	I have a lot of fun.	1	2	3	4	5	6
**C24.**	I get angry easily.	1	2	3	4	5	6
**C25.**	I am interested in many things.	1	2	3	4	5	6
**C26.**	I enjoy hurting others.	1	2	3	4	5	6
**C27.**	I don't quit a task before it is finished.	1	2	3	4	5	6
**C28.**	I am usually active and full of energy.	1	2	3	4	5	6
**C29.**	I cry easily.	1	2	3	4	5	6
**C30.**	I love to think up new ways of doing things.	1	2	3	4	5	6
**C31.**	I trust others.	1	2	3	4	5	6
**C32.**	I start tasks right away.	1	2	3	4	5	6
**C33.**	I make friends easily.	1	2	3	4	5	6
**C34.**	I like to solve complex problems.	1	2	3	4	5	6
**C35.**	I easily apologize when I have been wrong.	1	2	3	4	5	6
**C36.**	I work hard.	1	2	3	4	5	6
**C37.**	I love excitement.	1	2	3	4	5	6
**C38.**	I often feel blue.	1	2	3	4	5	6
**C39.**	I worry about what people think of me.	1	2	3	4	5	6

		Complete-ly false	Mostly false	Some-what false	Some-what true	Mostly true	Complete-ly true
**C40.**	I like to read.	1	2	3	4	5	6
**C41.**	I love to help others.	1	2	3	4	5	6
**C42.**	I think good manners are very important.	1	2	3	4	5	6

The following questions are about your everyday life and how you have been dealing with it since the start of the coronavirus epidemic in spring 2020.

D1. Do you face a heightened risk of contact with COVID-19 infected people due to your profession?

1 No

2 Yes, I have worked as a doctor, nurse or pharmacist

3 Yes, I have worked as a caregiver

4 Yes, I have worked as a police officer

5 Yes, I have been a service worker

6 Yes, I have worked as a public transport driver

7 Yes, I have worked as a teacher

8 Yes, other. Please specify: ………………………………….……………………………

D2. Do you feel you have been subject to unfavourable, negative or prejudiced attitude due to your possible contact with coronavirus?

1 No 2 Yes

D3. Have you been diagnosed with coronavirus?

1 No, I have not been diagnosed with coronavirus → *Proceed to question D5*

2 Yes, I have been diagnosed with coronavirus

D4. What symptoms did you have following the positive test result?

1 No symptoms (asymptomatic)

2 Mild or moderate symptoms (e.g. fever, fatigue, cold, cough, sore throat, muscle pains, changes in the sense of smell and taste)

3 Serious symptoms (e.g. long-term high fever, breathing difficulties, difficulties with speaking or walking) not requiring hospital treatment

4 I needed to go to the hospital

D5. **Have you been vaccinated against COVID-19?** Choose “yes” if you have received at least one vaccine dose.

1 No   2 Yes → *Proceed to question D7*

D6. **Are you planning to get vaccinated against COVID-19?**

1 Yes, definitely
2 I haven’t decided but probably yes
3 I don’t know
4 I haven’t decided but probably not
5 Definitely not

D7. **Have you noticed any changes in your mental health since the start of the coronavirus crisis compared to the time before the crisis?**

1 My mental health has declined
2 My mental health has remained unchanged
3 My mental health has improved
4 I don’t know

Please rate how much the following measures to prevent the spread of coronavirus currently in use and the characteristics of the situation have caused you stress.

		Not applicable	Caused no stress	Caused some stress	Caused significant stress
**D8.**	Recommended / required mask wearing in public spaces	1	2	3	4
**D9.**	Checking COVID health certificates (corona passports)	1	2	3	4
**D10.**	Ambiguity around restrictions and recommendations	1	2	3	4
**D11.**	Short notice for announcing or removing restrictions	1	2	3	4
**D12.**	Ambiguities around the vaccination schedule	1	2	3	4
**D13.**	Lack of clarity around when the pre-Covid situation would return	1	2	3	4
**D14.**	Risk of falling seriously ill due to the corona virus	1	2	3	4

		Not appli-cable	Caused no stress	Caused some stress	Caused significant stress
**D15.**	Risk of a person close to me falling seriously ill due to the corona virus	1	2	3	4
**D16.**	Risk that I will cause someone fall seriously ill due to the corona virus	1	2	3	4

D17. How stressed do you currently feel due to the coronavirus crisis?

1 Not at all

2 A little

3 Somewhat

4 A lot

5 Very much

D18. What have you done <u>in the past four (4) weeks</u> to prevent yourself or others from becoming infected with coronavirus? Select all of the measures you have taken.

1 Regularly washing and disinfecting your hands

2 Covering your mouth and nose when coughing or sneezing

3 Wearing a mask or a visor

4 Keeping a safe distance from others

5 Avoiding events and gatherings

6 Avoiding shopping centres and grocery stores

7 Staying home at any sign of illness

8 Getting tested for coronavirus

9 Getting vaccinated against coronavirus

10 Avoiding public transport

11 Avoiding indoor public spaces

12 Staying at home

13 None of the above

14 Other. Please specify: ……………………………………………………………..………………

To what extent have the following measures helped you deal with the coronavirus crisis <u>during the last twelve (12) months</u>?

		Never used them	Did not help	Helped somewhat	Helped significantly
**E1.**	Seeking help from people close to me				
**E2.**	Watching and listening to useful television and radio broadcasts or participating in webinars	1	2	3	4
**E3.**	Looking up additional information about my concerns online	1	2	3	4
**E4.**	Phoning helplines (such as the 1227 crisis helpline and the mental health helpline)	1	2	3	4
**E5.**	Seeing my primary care doctor (GP)	1	2	3	4
**E6.**	Using mental health web or phone apps	1	2	3	4
**E7.**	Seeing a psychiatrist, psychologist or psychotherapist	1	2	3	4
**E8.**	Talking to a priest or clergyman	1	2	3	4
**E9.**	Using different social services (such as personal care or home care to deliver medication and groceries or support at home)	1	2	3	4
**E10.**	Other. Please specify: ……………..………………………………	1	2	3	4

E11. Which of the following forms of mental health services do you feel are currently most lacking? Select all applicable responses.

1 Primary care doctors (GPs)

2 Psychiatrist’s consultations

3 Psychologist's or counsellor's consultations (including psychotherapy, counselling and family therapy)

4 Online or phone consultations with doctors, [mental health] nurses or counsellors

5 Crisis helplines

6 Victim support services

7 Pastoral care

8 Mental health websites listing help measures

9 Other. Please specify: ……………………………………………………………...…….

10 None of the above

Section F was only presented in web survey.

Earlier we asked you to rate your own health overall. Now we are interested in how you would rate the health of other people your age, based on the following descriptions.

F1.[a] **Leonard feels exhausted several days a week. He has trouble bending, lifting, and climbing stairs, and every day experiences pain that limits many of his daily activities. In the past year, he spent a few nights in a hospital, and over a week in bed due to illness.**

How would you assess Leonard's health?

1 Very good
2 Good
3 Average
4 Poor
5 Very poor

F2.[a] **Linda is usually energetic, but occasionally feels fatigued. She has some trouble bending, lifting, and climbing stairs. Her occasional pain does not affect her daily activities. In the past year, she spent a few days in bed due to illness.**

How would you assess Linda's health?

1 Very good
2 Good
3 Average
4 Poor
5 Very poor

F3.[a] **About once a week, Karen has no energy. She has some trouble bending, lifting, and climbing stairs, and each week experiences pain that limits some of her daily activities. In the past year, she spent a week in bed due to illness.**

How would you assess Karen's health?

1 Very good
2 Good
3 Average
4 Poor
5 Very poor

F4.[a] **Peter is energetic, and has little trouble with bending, lifting, and climbing stairs. He rarely experiences pain, except for minor headaches. In the past year Peter spent one day in bed due to illness.**

How would you assess Peter's health?

1 Very good
2 Good
3 Average
4 Poor
5 Very poor

Please read the following descriptions and rate how much of a problem does each described person have with feeling sad, low, or depressed.

F5.[a] Jan feels nervous and anxious. He worries and thinks negatively about the future, but feels better in the company of people or when doing something that really interests him. When he is alone he tends to feel useless and empty.

How much of a problem does Jan have with feeling sad, low, or depressed?

1 None
2 Mild
3 Moderate
4 Severe
5 Extreme

F6.[a] Henriette enjoys her work and social activities and is generally satisfied with her life. She gets depressed every 3 weeks for a day or two and loses interest in what she usually enjoys but is able to carry on with her day to day activities.

How much of a problem does Henriette have with feeling sad, low, or depressed?

1 None
2 Mild
3 Moderate
4 Severe
5 Extreme

F7.[a] Ken loves life and is happy all the time. He never worries or gets upset about anything and deals with things as they come.

How much of a problem does Ken have with feeling sad, low, or depressed?

1 None
2 Mild
3 Moderate
4 Severe
5 Extreme

F8.[a] Roberta feels depressed most of the time. She weeps frequently and feels hopeless about the future. She feels that she has become a burden on others and that she would be better dead.

How much of a problem does Roberta have with feeling sad, low, or depressed?

1 None
2 Mild
3 Moderate
4 Severe
5 Extreme

Today's date [b] |__|__| |__|__| 2021
Day Month

You have now reached the end of the questionnaire. Please make sure that you have answered all the questions.

Thank you very much for taking the time to complete the questionnaire!

Do you wish to be entered in the gift voucher prize draw? 1 No 2 Yes

If you have any additional information that you would like to share with us, please do so in the space below.

[a] Only in web survey, displayed to random subsamples
[b] Only in postal survey
