# Supplementary material for: Estonian National Mental Health Study: Design and methods for a registry‐linked longitudinal survey
Source: Brain Behav. 2023 Jun 5;13(8):e3106. doi: 10.1002/brb3.3106 (PMC10454261; doi:10.1002/brb3.3106)
Supplement: Supplementary file 5 — Additional file 5. Wave 3 questionnaire for minors (PDF) [file BRB3-13-e3106-s003.pdf]

ESTONIAN NATIONAL MENTAL HEALTH STUDY: WAVE 3 QUESTIONNAIRE FOR MINORS

Thank you for agreeing to participate in the third wave of the Estonian National Mental Health Study. The following questionnaire is about various aspects of your well-being. **To choose an answer option, circle the number next to the appropriate option or write it in the designated space. Every response is very important to us.** We assure that all your answers are kept safe and are not linked back to you.

First, we will ask you some questions about your general life situation.

A1. Sex 1 Male 2 Female

A2. Age as of today |__|__| years

A3. Ethnicity
1 Estonian
2 Russian
3 Other. Please specify: …………..…………………………...….

A4. What is the main language you speak at home?
1 Estonian
2 Russian
3 Other. Please specify: ………………….……………………….……..…………..…...…

A5. Who do you live with most days of the week? Select all applicable responses.
1 Mother
2 Father
3 Stepfather or mother's partner
4 Stepmother or father's partner
5 Sister(s)
6 Brother(s)
7 Stepsister(s)
8 Stepbrother(s)
9 Grandmother
10 Grandfather
11 My partner, boyfriend, girlfriend
12 Relatives (for example, uncle, aunt)
13 Friend(s)
14 Dormitory / rental apartment room-mate(s)
15 I live alone
16 I live in a foster family, family house or substitute home
17 Other. Please specify: ....

A6. How many siblings living in the same family as you do you have? Please specify the number of older and younger siblings. If you have no siblings, write 0 on both lines.

…… older siblings

…… younger siblings

A7. Are you a student?

1 Yes, in middle school

2 Yes, in high school

3 Yes, in vocational school

4 No

A8. Are you currently engaged in paid work?

1 Yes

2 No

A9. What is the current financial situation of your family?

1 We have money to spare

2 We have enough money to get by

3 We are barely making ends meet

4 We do not have enough money to cover unavoidable costs (such as utility bills)

5 Cannot say

A10. What is your current financial situation?

1 I have money to spare

2 I have enough money to get by

3 I am barely making ends meet

4 I do not have enough money to cover unavoidable costs

A11. Do you have your own room?

1 Yes

2 No, I share a room with my sibling(s)

3 No, I share a room with someone else

A12. Do you have a space in your home where you can be undisturbed?

1 Yes

2 No

A13. Where do you reside most days of the week?

1 Harju County, excluding Tallinn

2 Tallinn

3 Hiiu County

4	Ida-Viru County	12	Saare County
5	Jõgeva County	13	Tartu County, excluding Tartu
6	Järva County	14	Tartu
7	Lääne County	15	Valga County
8	Lääne-Viru County	16	Viljandi County
9	Põlva County	17	Võru County
10	Pärnu County	18	I do not reside in Estonia. Please specify your country of residence: ....
11	Rapla County		

A14. What type of settlement do you currently reside in?

1 A settlement with a population of less than 1000 or in the countryside

2 A settlement with a population of 1000–10,000

3 A settlement with a population of more than 10,000

The following questions are about your well-being, how you feel and your mental health.

Please rate your satisfaction with the following aspects of your life:

		Very satisfied	Quite satisfied	Not particularly satisfied	Not satisfied at all	
**B1.**	Life in general	1	2	3	4	
**B2.**	Your financial situation	1	2	3	4	
**B3.**	Family relations	1	2	3	4	
**B4.**	Friendships	1	2	3	4	
**B5.**	School and studies	1	2	3	4	5 I do not study

For the following questions, please indicate the answer that best describes your behaviour and how you have felt <u>over the past six (6) months</u>.

		Never	Rarely	Some-times	Often	Very often
**B6.**	How often do you struggle to complete a task once the more exciting parts of it are completed?	1	2	3	4	5
**B7.**	How often do you struggle with tasks that require systematisation or organisation?	1	2	3	4	5

		Never	Rarely	Some-times	Often	Very often
**B8.**	How often do you have problems with remembering meetings or responsibilities?	1	2	3	4	5
**B9.**	How often do you avoid or postpone activities or tasks that demand great mental effort?	1	2	3	4	5
**B10.**	How often do you start fidgeting or moving your arms/legs unintentionally if you have to sit in one place for a long time?	1	2	3	4	5
**B11.**	How often do you feel overactive and restless as if you have been ‘wound up’?	1	2	3	4	5

Carefully read the following list of problems and complaints that people sometimes experience. Please indicate how much each one has bothered you <u>during the last four (4) weeks</u>.

		Not at all	Rarely	Some-times	Often	Constantly
**B12.**	Sadness	1	2	3	4	5
**B13.**	Lack of interest in things	1	2	3	4	5
**B14.**	Feeling of worthlessness	1	2	3	4	5
**B15.**	Self-accusations	1	2	3	4	5
**B16.**	Recurrent thoughts of death or suicide	1	2	3	4	5
**B17.**	Feeling lonely	1	2	3	4	5
**B18.**	Hopelessness about the future	1	2	3	4	5
**B19.**	Inability to feel joy	1	2	3	4	5
**B20.**	Feeling easily irritated or annoyed	1	2	3	4	5
**B21.**	Feeling anxious or fearful	1	2	3	4	5
**B22.**	Feeling tense or unable to relax	1	2	3	4	5
**B23.**	Excessive worry about several things	1	2	3	4	5
**B24.**	Feeling so anxious or restless that it is hard to sit still	1	2	3	4	5
**B25.**	Being easily startled	1	2	3	4	5

		Not at all	Rarely	Some-times	Often	Constantly
**B26.**	Sudden panic attacks with palpitations, shortness of breath, faintness or other distressing bodily sensations	1	2	3	4	5
**B27.**	Fear of being away from home alone	1	2	3	4	5
**B28.**	Feeling afraid in public spaces or on the street	1	2	3	4	5
**B29.**	Fear of fainting in public	1	2	3	4	5
**B30.**	Fear of travelling by bus, tram, train or car	1	2	3	4	5
**B31.**	Fear of being the centre of attention	1	2	3	4	5
**B32.**	Fear of interacting with strangers	1	2	3	4	5
**B33.**	Fatigue or loss of energy	1	2	3	4	5
**B34.**	Diminished attention span or ability to concentrate	1	2	3	4	5
**B35.**	Resting does not restore strength	1	2	3	4	5
**B36.**	Being easily fatigued	1	2	3	4	5
**B37.**	Difficulty falling asleep	1	2	3	4	5
**B38.**	Restless or disturbed sleep	1	2	3	4	5
**B39.**	Waking up too early (spontaneously)	1	2	3	4	5
**B40.**	Deliberate self-harm (such as intentionally cutting your skin or causing pain, hitting yourself)	1	2	3	4	5

How much (or how often) have the following problems or complaints bothered you <u>during the last four (4) weeks?</u>

		Not at all	Rarely	Some-times	Often	Constantly
**B41.**	Sleeping less than usual, but still have a lot of energy	1	2	3	4	5
**B42.**	Starting lots more projects than usual or doing more risky things than usual	1	2	3	4	5

		Not at all	Rarely	Some-times	Often	Constantly
**B43.**	Unexplained aches and pains (e.g., head, back, joints, abdomen, legs)	1	2	3	4	5
**B44.**	Feeling that your illnesses are not being taken seriously enough	1	2	3	4	5
**B45.**	Hearing things other people couldn't hear, such as voices even when no one was around	1	2	3	4	5
**B46.**	Feeling that someone could hear your thoughts, or that you could hear what another person was thinking	1	2	3	4	5
**B47.**	Problems with memory (e.g., learning new information) or with location (e.g., finding your way home)	1	2	3	4	5
**B48.**	Unpleasant thoughts, urges, or images that repeatedly enter your mind	1	2	3	4	5
**B49.**	Feeling driven to perform certain behaviors or mental acts over and over again	1	2	3	4	5
**B50.**	Feeling detached or distant from yourself, your body, your physical surroundings, or your memories	1	2	3	4	5

How much do the following statements apply to you? Please select the most applicable answer.

		Completely false	Mostly false	Neither true nor false	Mostly true	Completely true
**B51.**	Most of the time I feel lively and energetic.	1	2	3	4	5
**B52.**	Most of the time I feel attentive and alert.	1	2	3	4	5
**B53.**	I am hopeful and enthusiastic about the future.	1	2	3	4	5

B54. Sometimes things happen to people that are particularly frightening or traumatic. Such events can include natural disasters and other catastrophes, wars, serious accidents and fires, a serious illness, being placed under intensive care, sexual or physical assault or abuse, witnessing a murder, suicide or injuries and the sudden death of someone close. **Have you ever experienced such events?**

1 No, never → *Proceed to question B59*

2 Yes, more than a month ago

3 Yes, less than a month ago

Below is a list of problems and complaints that people sometimes have in response to stressful experiences. Please indicate how much each problem has bothered you <u>during the last four (4) weeks.</u>

		Not at all	Rarely	Some-times	Often	Constantly
**B55.**	Repeated, disturbing memories, thoughts or images of a stressful experience	1	2	3	4	5
**B56.**	Feeling very upset when something reminded you of a stressful experience	1	2	3	4	5
**B57.**	Avoiding activities or situations because they reminded you of a stressful experience	1	2	3	4	5
**B58.**	Being watchful or easily startled	1	2	3	4	5

Next, we want to know the importance of food and eating in your life <u>over the past three (3) months</u>.

B59. Have you spent a considerable amount of time thinking about food and your weight?

1 No 2 Yes

B60. Have you considerably limited your diet <u>over the past three months</u>?

1 No 2 Yes

B61. Have you been binge eating (eating more than usual) <u>over the past three months</u>?

1 No → *Proceed to question B63* 2 Yes

B62. During these binges, have you felt that you cannot control your eating?

1 No 2 Yes

B63. Have you deliberately made yourself vomit, used laxatives or appetite suppressants to control your weight <u>over the past three months</u>?

1 No 2 Yes

Carefully read the following statements and indicate to what extent you agree with each statement.

		Completely agree	Rather agree	Rather disagree	Completely disagree
**B64.**	I am fascinated by dates.	1	2	3	4
**B65.**	I usually notice car number plates or similar strings of information.	1	2	3	4
**B66.**	I find it easy to "read between the lines" when someone is talking to me.	1	2	3	4
**B67.**	I find social situations easy.	1	2	3	4
**B68.**	If I try to imagine something, I find it very easy to create a picture in my mind.	1	2	3	4
**B69.**	It does not upset me if my daily routine is disturbed.	1	2	3	4
**B70.**	When I'm reading a story, I can easily imagine what the characters might look like.	1	2	3	4
**B71.**	I find it difficult to work out people's intentions.	1	2	3	4
**B72.**	I find it hard to make new friends.	1	2	3	4
**B73.**	I enjoy doing things spontaneously.	1	2	3	4

In the following section we will be asking about your general health and health behaviour.

C1. How tall are you? (without shoes) ……… cm

C2. How much do you weigh? (without clothes) If you are pregnant, note your weight prior to your pregnancy.

……… kg

C3. How would you assess your current state of health?

1 Very good
2 Good
3 Average
4 Poor
5 Very poor

C4. Do you have any long-standing (chronic) illness or health problem?

1 No
2 Yes

C5. How concerned have you been about your health <u>over the past six (6) months</u>?

1 Not at all
2 A little
3 To some extent
4 A lot
5 Very much

C6. <u>Over the past three (3) months</u>, how often in your leisure time have you been active (playing sports, doing gardening, high-speed cycling or brisk walking, etc.) for at least 30 min at a time so that you are slightly out of breath or sweating?

1 Never
2 Once a month or less
3 2–3 times per month
4 Once a week
5 2–3 times a week
6 4–6 times a week
7 Every day

C7. How many minutes do you walk or ride a bicycle on a regular day?

1 Less than 15 minutes a day
2 15–30 minutes a day
3 30–60 minutes a day
4 More than 60 minutes a day

C8. Have you smoked <u>in the past three (3) months</u>?

1 No → **C9a. Have you ever smoked?**

 1 I have never smoked
 2 I quit more than 6 months ago
 3 I quit less than 6 months ago

2 Yes → **C9b. What characterises your smoking?** Select all applicable responses.

 1 I smoke cigarettes/cigars/a pipe daily
 2 I smoke e-cigarettes or other smoke-free products daily
 3 I smoke cigarettes/cigars/a pipe occasionally
 4 I smoke e-cigarettes or other smoke-free products occasionally

C10. How often have you had a drink containing alcohol <u>over the past three (3) months</u>?

1 Never → *Proceed to question C13*
2 Monthly or less
3 2–4 times per month
4 2–3 times per week
5 4 or more times per week

C11. How many units of alcohol did you usually consume at one time <u>in the past three (3) months</u>?

1 1–2
2 3–4
3 5–6
4 7–9
5 10+

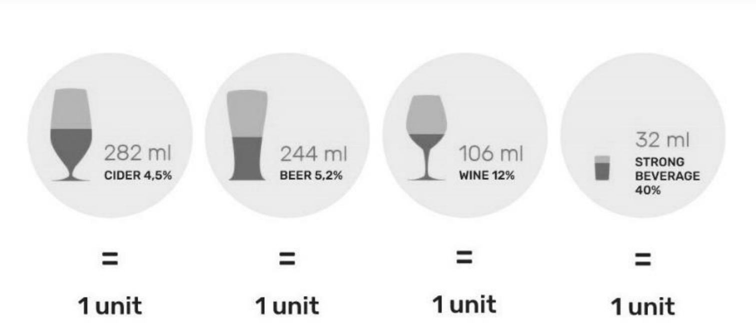

C12. How often have you had 6 or more units on a single occasion?

1 Never
2 Less than once a month
3 Once a month
4 Once a week
5 Every day or almost every day

C13. How often have you used narcotic substances <u>over the past three (3) months</u>? Narcotic substances include cannabis, cocaine or crack, 'party drugs' (such as ecstasy), hallucinogens (such as LSD), heroine, solvents or inhalants (such as glue) or methamphetamine (such as speed).

1 Never
2 Once a month or less
3 2–4 times a month
4 2–3 times a week
5 4 or more times a week

C14. On average, how many hours a day have you slept <u>in the past three (3) months</u>? Include naps in your calculation.

…… hours and …… minutes on a weekday

…… hours and …… minutes at the weekend

The following questions are about your emotions and how you have dealt with them <u>over the past three (3) months</u>.

C15. How often have you recently felt that difficulties were piling up so high that you could not overcome them?

1 Very rarely
2 Quite rarely
3 Sometimes
4 Quite often
5 Very often

Please rate how often the following statements apply to you.

C16. When I am upset, I have difficulty controlling my behaviours.

1 Almost never
2 Sometimes
3 Often
4 Most of the time
5 Almost always

C17. When I feel low-spirited, I only think about my problems and I find it hard to focus on anything else.

1 Almost never
2 Sometimes
3 Often
4 Most of the time
5 Almost always

C18. When I am upset, I believe that there is nothing I can do to make myself feel better.

1 Almost never
2 Sometimes
3 Often
4 Most of the time
5 Almost always

Next, we will ask you to respond to questions about your relationships with your family and friends. Please rate how much the following statements apply to you.

C19. If I have a worry, I feel comfortable talking to my family members about it.

1 Completely disagree
2 Tend to disagree
3 Tend to agree
4 Completely agree

C20. I can rely on my family members for help if something bad happens to me.

1 Completely disagree
2 Tend to disagree
3 Tend to agree
4 Completely agree

C21. I can rely on my friends for help if something bad happens to me.

1 Completely disagree
2 Tend to disagree
3 Tend to agree
4 Completely agree

C22. How often does emotional abuse (swearing or yelling at you, insulting, etc.) occur in your family?

1 Never
2 Very rarely
3 It has occurred occasionally
4 Quite often
5 Almost every day

C23. Have you ever experienced sexual abuse or unwanted sexual contact?

1 Never
2 Yes, but not within the last 12 months
3 Yes, within the last 12 months
4 Don’t wish to say

C24. Have you ever experienced physical abuse?

1 Never
2 Yes, but not within the last 12 months
3 Yes, within the last 12 months
4 Don’t wish to say

C25. How often have you been bullied <u>in the past couple of months</u>? *Bullying refers to one person purposefully and repeatedly making another person feel bad and the victim finds it hard to defend themselves. Disputes and disagreements are not bullying, neither are teasing and tussling.

1 It has not happened at all → *Proceed to question C27*
2 Once or twice
3 2 or 3 times a month
4 About once a week
5 Several times a week

C26. Where have you been bullied <u>in the past couple of months</u>? Select all applicable responses.

1 At school
2 At home
3 In a sports club
4 Other. Please specify: ……………………………………………………………………

C27. How often have you taken part in bullying another person <u>in the past couple of months</u>?

1 It has not happened at all
2 Once or twice
3 2 or 3 times a month
4 About once a week
5 Several times a week

C28. How often have you seen someone else being in bullied <u>in the past couple of months</u>?

1 It has not happened at all
2 Once or twice
3 2 or 3 times a month
4 About once a week
5 Several times a week

C29. How often have you been cyber-bullied <u>in the past couple of months</u>? (e.g., someone sent mean instant messages, wall postings, emails and text messages; created a website making fun of you; posted unflattering or inappropriate pictures of you online without permission or shared them with others)

1 It has not happened at all
2 Once or twice
3 2 or 3 times a month
4 About once a week
5 Several times a week

The following questions are about using smart devices and social media in your everyday life.

C30. How many hours on a regular day do you use electronic devices (computer, tablet, smartphone, etc.) for (school)work?

1 not at all
2 less than one hour a day
3 1–2 hours a day
4 2–4 hours a day
5 4–6 hours a day
6 more than 6 hours a day

C31. How many hours on a regular day do you use electronic devices (TV, computer, tablet, smartphone, etc.) for leisure activities (incl. YouTube and games)?

1	not at all	4	2–4 hours a day
2	less than one hour a day	5	4–6 hours a day
3	1–2 hours a day	6	more than 6 hours a day

C32. How many hours on a regular day do you use electronic devices (computer, tablet, smartphone, etc.) for communication outside school or work?

1	not at all	4	2–4 hours a day
2	less than one hour a day	5	4–6 hours a day
3	1–2 hours a day	6	more than 6 hours a day

The following questions are about your social media use.

C33. Do you use social media? (e.g. Facebook, Twitter, Instagram)

1 no → *proceed to question C38*

2 yes

		Never	Rarely	Some-times	Often	Very often
**C34.**	How often do you find that you spend more time with social media than you intended?	1	2	3	4	5
**C35.**	How often have you neglected your obligations or chores because of using social media?	1	2	3	4	5
**C36.**	How often do you feel preoccupied with using social media?	1	2	3	4	5
**C37.**	How often have people told you that you use social media too much?	1	2	3	4	5

If A7=4, then C38-C48 are skipped.

Now we are going to ask about your school and studies. Please indicate how much you agree or disagree with each statement.

		Completely agree	Rather agree	Rather disagree	Completely disagree
**C38.**	I do well at school.	1	2	3	4
**C39.**	I like distance learning.	1	2	3	4
**C40.**	My family shows interest in my school work.	1	2	3	4

		Completely agree	Rather agree	Rather disagree	Completely disagree
**C41.**	My teachers show interest in my school work.	1	2	3	4
**C42.**	I am able to regulate my learning.	1	2	3	4
**C43.**	I can focus on a task when studying independently.	1	2	3	4
**C44.**	I can monitor my learning progress.	1	2	3	4
**C45.**	I'm able to pick my study methods.	1	2	3	4
**C46.**	I easily get over failures related to school work and try again.	1	2	3	4
**C47.**	I know my strengths and weaknesses.	1	2	3	4
**C48.**	I know how to reach my goals.	1	2	3	4

The following questions are about your everyday life and how you have been dealing with it <u>since the start of the coronavirus epidemic in spring 2020</u>.

D1. Have you been tested for coronavirus?

1 No → *Proceed to question D4*

2 Yes

D2. Have you been diagnosed with coronavirus?

1 No, I have not been diagnosed with coronavirus → *Proceed to question D4*

2 Yes, I have been diagnosed with coronavirus

D3. What symptoms did you have following the positive test result?

1 No symptoms (asymptomatic)

2 Mild or moderate symptoms (e.g. fever, fatigue, cold, cough, sore throat, muscle pains, changes in the sense of smell and taste)

3 Serious symptoms (e.g. long-term high fever, breathing difficulties, difficulties with speaking or walking) not requiring hospital treatment

4 I needed to go to the hospital

D4. Have you been vaccinated against COVID-19? *Choose "yes" if you have received at least one vaccine dose.*

1 No

2 Yes → *Proceed to question D6*

D5. Are you planning to get vaccinated against COVID-19?

1 Yes, definitely

2 I haven’t decided but probably yes

3 I don’t know

4 I haven’t decided but probably not

5 Definitely not

Please rate how much the following measures to prevent the spread of coronavirus currently in use have caused you stress.

		Not applicable	Caused no stress	Caused some stress	Caused significant stress
**D6.**	Recommended / required mask wearing in public spaces	1	2	3	4
**D7.**	Checking COVID health certificates (corona passports)	1	2	3	4

D8. H**ow stressed do you currently feel due to the coronavirus crisis?**

1 Not at all

2 A little

3 Somewhat

4 A lot

5 Very much

D9. What have you done in the past four (4) weeks to prevent yourself or others from becoming infected with coronavirus? Select all of the measures you have taken.

1 Regularly washing and disinfecting your hands

2 Covering your mouth and nose when coughing or sneezing

3 Wearing a mask or a visor

4 Keeping a safe distance from others

5 Avoiding events and gatherings

6 Avoiding shopping centres and grocery stores

7 Staying home at any sign of illness

8 Getting tested for coronavirus

9 Getting vaccinated against coronavirus

10 Avoiding public transport

11 Avoiding indoor public spaces

12 Staying at home

13 None of the above

14 Other. Please specify: ……………………………………………………….………………

To what extent have the following measures helped you deal with the coronavirus crisis <u>during the last twelve (12) months</u>?

		Never used them	Did not help	Helped somewhat	Helped significantly
**E1.**	Seeking help from people close to me	1	2	3	4
**E2.**	Watching and listening to useful television and radio broadcasts or participating in webinars	1	2	3	4
**E3.**	Looking up additional information about my concerns online	1	2	3	4
**E4.**	Phoning helplines (such as the 1227 crisis helpline and the mental health helpline)	1	2	3	4
**E5.**	Seeing my primary care doctor (GP)	1	2	3	4
**E6.**	Using mental health web or phone apps	1	2	3	4
**E7.**	Seeing a psychiatrist, (school) psychologist or psychotherapist	1	2	3	4
**E8.**	Talking to a priest or clergyman	1	2	3	4
**E9.**	Other. Please specify: …………………..…………………………	1	2	3	4

E10. Which of the following forms of mental health services do you feel are currently most lacking? Select all applicable responses.

1 Primary care doctors (GPs)

2 Psychiatrist’s consultations

3 (School) psychologist's or counsellor's consultations (including psychotherapy, counselling and family therapy)

4 Online or phone consultations with doctors, (mental health) nurses or counsellors

5 Crisis helplines

6 Victim support services

7 Pastoral care

8 Mental health websites listing help measures

9 Other. Please specify: ……………………………………………………………...…….

10 None of the above

You have now reached the end of the questionnaire. Please make sure that you have answered all the questions.

Thank you very much for taking the time to complete the questionnaire!

If you have any additional information that you would like to share with us, please do so in the space below.
