# Supplementary material for: Estonian National Mental Health Study: Design and methods for a registry‐linked longitudinal survey
Source: Brain Behav. 2023 Jun 5;13(8):e3106. doi: 10.1002/brb3.3106 (PMC10454261; doi:10.1002/brb3.3106)
Supplement: Supplementary file 6 — Additional file 6. Methods of the validation study (PDF) [file BRB3-13-e3106-s002.pdf]

ESTONIAN NATIONAL MENTAL HEALTH STUDY

Methods of the validation study

Participants

A subsample (n=3698) of wave 1 survey respondents was invited to participate in the validation study carried out during the second survey wave. The eligibility criteria for the validation study were a) having completed wave 1 survey online, b) a good command of Estonian, c) a valid e-mail address, and d) possibility to use smartphones with mobile internet access. The subsample was further divided into ecological momentary assessment (EMA) subsample who completed only the EMA survey, and a sleep and physical activity (SPA) subsample who also wore an activity monitor to assess sleep time and physical activity and donated saliva samples for cortisol assessment.

Due to logistic reasons (e.g., participants had to receive activity monitors and saliva kits and instructions personally), only a limited number of persons were included in the SPA subsample. It was determined that 200 participants could be tested within two 1-week measurement periods. For feasibility, only persons living in selected areas (cities of Tallinn and Tartu, and Harju, Rapla, Tartu and Põlva counties) could be enrolled. To keep gender balance, an equal number of men and women were included from each area for the subsample. Counting on a participation rate of 1/3, 600 persons were initially invited to attend the SPA substudy; however, after a lower-than-expected consent rate in 2 weeks, 400 additional invitations were sent. All the remaining eligible persons (meeting the eligibility criteria but not residing in selected areas or not included in the SPA subsample; n = 2698) were included in the EMA subsample.

Measures

The 16-question Emotion regulation strategies questionnaire (ERS; Table 1) was used as pre- and post-study questionnaire. Participants filled out the ERS three times – one day before the EMA study, immediately after the study and two weeks after the study. The EMA questionnaire (Table 2) of affective experiences, the context of these emotions and affect regulation strategies used to cope with the situations was filled out five times a day. Once a day, participants were asked to report on their bedtime and sleep quality, physical activity, alcohol intake and general health assessment.

In the SPA substudy, participants were asked to wear GeneActiv monitor for the study period for objective measures of physical activity (1) and sleep (2). Additionally, salivary cortisol samples were collected on four occasions during the SPA substudy using Salivette® Cortisol (SARSTEDT, Numbrecht, Germany; https://www.sarstedt.com) saliva collection kits.

Procedures

Stage 1. Enrollment for the validation study took place in 04.05.2021–28.06.2021. The invitation with personalized study link was sent via e-mail and also displayed at the end of the wave 2 web survey to eligible persons. The personalized link directed eligible persons to the *formr* platform (https://formr.org; 3) where they were informed about the study details, asked to give their informed consent and leave their contact information. The SPA subsample was also asked whether they were willing to donate salivary samples. After registration, participants could familiarize themselves with the commented EMA questionnaire. If eligible persons had neither given their consent nor declined to participate in the validation study, up to two reminders were sent via e-mail two and three weeks after the initial invitation.

Stage 2. For each participant, the study started the following week after the registration. The EMA subsample received SMS reminder instructing them to fill out the ERS questionnaire for the first time a day before the study. The SPA subsample was contacted up to a week before the beginning of the study to arrange the delivery and pick-up of the GeneActiv monitor and the saliva collection kit of four

sampling tubes. The participants were asked to wear the monitor as often as they could during the study (ideally, 24 hours a day, for seven days).

Stage 3. During this stage, participants were asked to fill out the EMA questionnaires for seven consecutive days. We used a signal-contingent design for our EMA validation studies, where participants were asked to fill out the questionnaire once they received an SMS on their smartphone (the prompt). There were five prompts per day, timed randomly one per 90-minute time slot between 9 am and 9 pm: 09.00–10.30, 11.30–01.00, 02.00–03.30, 04.30–06.00, 07.00–08.30. Each prompt was open for an hour. In the SPA substudy, participants took four salivary samples on the two last days of the study. Two samples were taken per day, one in the morning 30–45 minutes after waking up and one in the evening between 8 pm and 9 pm.

Stage 4. After the last prompt of the week participants were asked to fill out the second ERS questionnaire. After that, participants were provided with feedback/summary on the emotions they had experienced and emotion regulation strategies they had used during the week. In the SPA substudy, the salivary samples and GENEActiv monitors were collected from the participants the following morning after the last prompt.

Stage 5. Two weeks after the end of the study participants received the last SMS which asked them to fill out the third ERS questionnaire.

Participants of the validation study were entered twice in the gift card draw after the second survey wave, thus increasing their chance of winning.

Table 1. Emotion regulation strategies questionnaire (ERS)

	Item	Response options				
1	I suppress my feelings	1	2	3	4	5
2	I act out my feelings	1	2	3	4	5
3	I resolve problems causing these feelings	1	2	3	4	5
4	I busy myself with something to take my mind off my troubles	1	2	3	4	5
5	I think about how things are not as bad as they initially seemed	1	2	3	4	5
6	I think about how bad things can be good for something	1	2	3	4	5
7	I accept that things are as they are	1	2	3	4	5
8	I do breathing or relaxation exercises	1	2	3	4	5
9	I am physically active or work out	1	2	3	4	5
10	I smoke	1	2	3	4	5
11	I consume alcohol or other drugs	1	2	3	4	5
12	I eat or snack on something	1	2	3	4	5
13	I eat more than I'd like	1	2	3	4	5
14	I seek emotional support from my loved ones	1	2	3	4	5
15	I seek professional help or support	1	2	3	4	5
16	I do something else: __________________________	1	2	3	4	5

Note. The instruction to the questionnaire was: *As follows, you will find a list of different strategies people use to cope with their negative feelings. Please indicate on each row how often you use this specific strategy. Never or hardly ever (1) – rarely (2) – occasionally (3) – often (4) – very often or always (5)*

Table 2. Ecological momentary assessment (EMA) questionnaire

	Item	Response options
	**At this moment, how strongly do you feel…** *Mark on each row how strongly do you currently experience one or more of these feelings*	
1	**joyful, excited**	Not at all ----- Very strongly (7-point scale)
2	**satisfied, relaxed**	Not at all ----- Very strongly (7-point scale)
3	**worried, anxious**	Not at all ----- Very strongly (7-point scale)
4	**sad, disappointed**	Not at all ----- Very strongly (7-point scale)
5	**irritated, angry**	Not at all ----- Very strongly (7-point scale)
6	**tense, stressed**	Not at all ----- Very strongly (7-point scale)
7	**tired, listless**	Not at all ----- Very strongly (7-point scale)
8	**What are Your current feelings related to?** *Choose all relevant options.*	a. An ongoing situation<br>b. An earlier situation<br>c. A situation in the future<br>d. Same situation I mentioned in the previous prompt<br>e. Work or studies<br>f. Spending leasure time<br>g. Close relationships<br>h. Other relationships<br>i. COVID-19<br>j. Celebrating something<br>k. Eating<br>l. Something else/ hard to say
9	**What have you tried to do to make yourself feel better?** *Mark all strategies you have used since the previous prompt.*	a. I haven't tried anything<br>b. I suppressed my feelings<br>c. I acted out my feelings<br>d. I solved problems causing these emotions<br>e. I busied myself with something to take my mind off my troubles<br>f. I thought how things are not as bad as they initially seemed<br>g. I thought about how bad things can be good for something<br>h. I accepted that things are as they are<br>i. I meditated or did relaxation exercises<br>j. I was physically active or worked out<br>k. I smoked<br>l. I consumed alcohol or other drugs<br>m. I ate or snacked on something<br>n. I ate more than I would have liked<br>o. I shought emotional support from my loved ones<br>p. I sought professional help or support<br>r. I did something else: _________
10	**Have these techniques made you feel any better?**	Not at all ----- A lot better (7-point scale)
11*	**How well did you sleep last night?**	Very unwell ----- Very well (7-point scale)
12*	**What time did you go to bed last night?**	[drop-down menu with 15-min intervals**]
13*	**What time did you wake up this morning?**	[drop-down menu with 15-min intervals**]
14*	**How many minutes did you engage in moderate to high physical activity yesterday?** *(f.e. practised sports, worked out or went for a walk so that it made you sweat or gasp lightly)*	Insert minutes ______
15*	**How many minutes did you engage in light physical activity yesterday?** *(f.e. slow-paced walk or cycling/bike ride)*	Insert minutes ______
16*	**Did you consume any alcoholic beverages yesterday?**	Yes / No
17*	**How do you assess your health at the moment?**	Very bad ------ Very good (7-point scale)

Note. * Items included only in the first prompt of each day. ** Options covered 15-min periods from 8 pm to 3 am for item 12, and from 5 am to 12 pm for item 13; for times outside these periods, it was possible to enter the exact time as free text.

References

1. Lin W-Y, Verma V, Lee M-Y, Lai C-S. Activity monitoring with a wrist-worn, accelerometer-based device. Micromachines. 2018;9:450. doi:10.3390/mi9090450
2. Van Hees VT, Sabia S, Jones SE, Wood AR, Anderson KN, Kivimäki M, et al. Estimating sleep parameters using an accelerometer without sleep diary. Sci Rep. 2018;8:12975. doi:10.1038/s41598-018-31266-z
3. Arslan RC, Walther MP, Tata CS. formr: A study framework allowing for automated feedback generation and complex longitudinal experience-sampling studies using R. Behav Res Methods. 2020;52:376–87. doi:10.3758/s13428-019-01236-y
